# Splicing of a non-coding antisense transcript controls *LEF1* gene expression

**DOI:** 10.1093/nar/gkv502

**Published:** 2015-05-18

**Authors:** Manuel Beltran, Estel Aparicio-Prat, Rocco Mazzolini, Alba Millanes-Romero, Pere Massó, Richard G. Jenner, Víctor M. Díaz, Sandra Peiró, Antonio García de Herreros

**Affiliations:** 1 Programa de Recerca en Càncer, Institut Hospital del Mar d'Investigacions Mèdiques, 08003 Barcelona, Spain; 2 UCL Cancer Institute, University College London, London, WC1E6BT, UK; 3 Departament de Ciències Experimentals i de la Salut, Universitat Pompeu Fabra, 08003 Barcelona, Spain

## Abstract

In this report we have analyzed the role of antisense transcription in the control of LEF1 transcription factor expression. A natural antisense transcript (NAT) is transcribed from a promoter present in the first intron of *LEF1* gene and undergoes splicing in mesenchymal cells. Although this locus is silent in epithelial cells, and neither NAT transcript nor *LEF1* mRNA are expressed, in cell lines with an intermediate epithelial-mesenchymal phenotype presenting low LEF1 expression, the NAT is synthesized and remains unprocessed. Contrarily to the spliced NAT, this unspliced NAT down-regulates the main *LEF1* promoter activity and attenuates *LEF1* mRNA transcription. Unspliced LEF1 NAT interacts with *LEF1* promoter and facilitates PRC2 binding to the *LEF1* promoter and trimethylation of lysine 27 in histone 3. Expression of the spliced form of *LEF1* NAT in *trans* prevents the action of unspliced NAT by competing for interaction with the promoter. Thus, these results indicate that *LEF1* gene expression is attenuated by an antisense non-coding RNA and that this NAT function is regulated by the balance between its spliced and unspliced forms.

## INTRODUCTION

Epithelial-to-Mesenchymal Transition (EMT) is a process in which epithelial cells lose epithelial characteristics and acquire a mesenchymal phenotype (1). This process is relevant during early phases of embryo development, such as gastrulation and neural crest delamination, as well as during tumor progression. At the molecular level, this process is characterized by the down-regulation of E-cadherin, a protein with a central role in the adherens junctions, and the up-regulation of mesenchymal markers, such Snail1, Zeb1/2, Fibronectin, Lymphoid Enhancer Factor 1 (LEF1) and Vimentin (1–3). During tumor progression, this process takes place through an intermediate or ‘epithelial metastable’ phenotype, co-expressing moderate levels of epithelial and mesenchymal genes (3,4). These intermediate phenotypes are particularly relevant since they combine epithelial and mesenchymal traits and facilitate cohort migration and metastasis (4). Among the genes induced during EMT, the transcription factor Snail1 plays a key role in this transition since it is rapidly induced, preceding the expression of the rest of the mesenchymal genes, is required for EMT and is capable of triggering this conversion when over-expressed in epithelial cells (2,3). For these reasons, Snail1 over-expression has been used as a method to impose the mesenchymal phenotype on epithelial cells, either with no expression of mesenchymal genes or with an intermediate morphology (3).

Natural antisense transcripts (NATs) are a type of non-coding RNAs usually located in the same locus as well characterized protein-coding genes (5). They are transcribed in the opposite direction to the mRNA of the coding gene and range in length from 0.5 to 10 kb. Global transcriptome analysis shows that up to 70% of the protein-coding transcripts have antisense counterparts and that modification of NAT levels alters the expression of the sense gene (6,7). NATs can work in *cis* or in *trans* (7,8) and in most cases promote the epigenetic silencing of the target genes (9–12). In this work, we have analyzed the expression of an annotated NAT (13) corresponding to LEF1. The *LEF1* gene encodes a transcriptional factor that cooperates with β-catenin in the expression of target genes (14) and its expression is up-regulated in mesenchymal cells (15,16). We have observed the expression in epithelial cells of a NAT overlapping the *LEF1* promoter. The data presented in this work indicate that this *LEF1* NAT exists as both spliced and unspliced forms and, in its unspliced form, represses *LEF1* gene expression by interacting with the *LEF1* promoter and recruiting the Polycomb Repressive Complex 2 to the *LEF1* promoter.

## METHODS AND MATERIALS

### Plasmid construction

Since *LEF1* gene contains several transcription start sites, the different constructs refer to the initiation codon of the full-length protein (NP_057353.1). The *LEF1* −1856/+58 promoter was cloned from HT-29 M6 genomic DNA, using high-fidelity Pfx Platinium polymerase (Invitrogen) and oligonucleotides corresponding to the sequences −1837/−1856 and +37/+58 that contained restriction sites for KpnI and SmaI enzymes. The *LEF1* NAT promoter +857/+66 was cloned using a oligonucleotide corresponding to +66/+82 and another one to +841/+857 provided with a HindIII site and digesting the fragment with HindIII. Both fragments were inserted in pGL3 in KpnI/SmaI or HindIII sites, respectively. pGL3 −1856/+857 was obtained by opening the plasmid pGL3 −1856/+56 with HindIII and cloning the +856/+57 amplification product in forward orientation. pGL3 −1856/+857 (Δ+370/+786) was obtained by cutting the plasmid pGL3 −1856/+857 with RsrII (position +370) and AgeI (position +786), followed by religation of the digested vector. The vector still retains the promoter region +786/+857.

The expression plasmids for *LEF1* NAT (unspliced) were obtained by inserting the +58/−1856 or +213/−1856 amplification products in the EcoRI site of pBabe-Puro (Addgene), or between KpnI and HindIII sites in pcDNA3 in the antisense direction. The different NAT deletions were constructed using oligonucleotides corresponding to the sequences −18/+1, −369/-387, −750/−769, −1439/−1459 as sense oligos and −1837/−1856, −1463/−1445, −879/−859 −405/−382 as reverse oligos. Fragments were inserted in the EcoRV site of pcDNA3 vector. All amplification products were obtained using high-fidelity Pfx Platinium polymerase (Invitrogen) and verified by sequencing.

Spliced NAT (+213/−8660) was amplified from SW-480 cells by reverse transcriptase-polymerase chain reaction (RT-PCR) using oligonucleotides corresponding to sequences +213/+195 and −8660/−8636 tagged with KpnI and XhoI sites, respectively and cloned in pcDNA3 digested with KpnI and XhoI. The PCR product was 517 bp long and was sequenced; it contains sequences corresponding to +213/−68, −5652/−5753 and −8523/−8660.

### RNA immunoprecipitation (RIP)

RIP assays were performed as previously described (17) without crosslinking. Cells were washed and then lysed with polysomal lysis buffer (100 mM KCl, 5 mM MgCl_2_, 10 mM Hepes pH 7, 0.5% NP-40, 1 mM DDT, 100 units/ml RNase out (Invitrogene)), supplemented with a protease inhibitor cocktail. When indicated, the cell extract (500 μg) was treated with 400 units of the nucleases DNase I, RNase H, RNase V1 or RNase A (Ambion), in a final volume of 1 ml. Cell extracts were pre-cleared with irrelevant IgGs, and protein G-magnetic beads previously blocked with salmon sperm (1 mg/ml), poly dI-dC (1 μg/ml), and BSA (100 μg/ml). After immunoprecipitation with specific mAbs or with an irrelevant immunoglobulin G (Sigma/DAKO), samples were purified with protein G-magnetic beads. After washing with NT2 Buffer (50 mM Tris pH 7.4, 150 mM NaCl, 1 mM MgCl_2_, 0.1% NP40 plus RNase and protease inhibitors), RNA was extracted using the Trizol (Invitrogen) method. Transcripts were analyzed by semi-quantitative or quantitative RT-PCR as indicated in Supplementary Methods.

### *In vivo* NAT/ *LEF1* promoter binding assays

Cells were transfected with *in vitro* synthesized biotinylated-NAT or irrelevant RNA and the *LEF1* promoter or *CDH1* promoter when indicated. Cells were crosslinked with formaldelhyde as described in the Supplemental Methods. Anti-biotin antibody (Sigma) and protein A-agarose were used to immunoprecipitate biotinylated RNAs. Samples were washed as for ChIP assays (18), treated with elution buffer (100 mM Na_2_CO_3_, 1% SDS) and purified as for ChIP assays. The presence of the amplicons −1806/−1628, −1306/−1188, −904/−703 (corresponding to *LEF1* promoter), +3864/+4048 (*LEF1* mRNA second intron) and +570/+744 (corresponding to Luciferase) were measured by qPCR. The sequence of this amplicon corresponds to +483/+657 with respect to the Luciferase translation start site. The presence of *CDH1* amplicon was also determined as negative control as reported (18).

### Biotinylated-oligonucleotide pull-down (BOPA) assays

Biotinylated-DNA pull-down assays (DNA-BOPA) were carried out using a biotinylated DNA fragment corresponding to the *LEF1* promoter (−1856/+58), generated by PCR using the corresponding DNA as template with the same specific oligonucleotides used for cloning, with a 5′-biotin label on the sense primer. HT-29 M6 cells were lysed with the polysomal lysis buffer (see RIP assays), and diluted in binding buffer (20 mM Hepes pH 7.6, 150 mM KCl, 3 mM MgCl_2_, 10% glycerol, 3 mg/ml BSA, 0.2 Triton X-100, 20 μg/ml poly dI-dC, 1 mM DTT, plus protease and RNase inhibitors). Pre-clearing was performed by incubating with protein G-agarose blocked with salmon sperm (1 mg/ml) and mouse IgG (10 μg/ml). After pre-clearing, samples (500 μg) were incubated for 4 h in binding buffer with the biotinylated probes (2 μg), *in vitro* synthesized RNA (4 μg), and antibodies against biotin (20 μg/ml) in a final volume of 1 ml. Samples were pulled-down with protein G-agarose, washed with binding buffer supplemented with 0.1% Tween-20 and analyzed by western blot.

RNA-BOPA was performed using biotinylated LEF1 NAT, generated by *in vitro* transcription adding biotin16-UTP (Ambion) to the reaction. The procedure was as described for DNA-BOPA.

### Triplex DNA analysis by electrophoresis mobility shift assay (EMSA)

The DNAs corresponding to the indicated *LEF1* promoter fragments were amplified by PCR. After synthesis, the fragments were ^32^P-labeled using [γ-32P]ATP and T4 polynucleotide kinase (New England Biolabs), and purified with Illustra MicroSpin G-50 columns (GE Healthcare). The amount of the incorporated radioactivity was measured by liquid scintillation counter. The RNAs corresponding to the NAT fragments were transcribed *in vitro* and purified as detailed above.

Each condition of the assay contained equal concentrations of the ^32^P-labeled DNA (50 000 cpm) and increasing concentrations of NAT, in a binding buffer consisting of 10 mM Tris-Borate, pH 7.2 and 10 mM MgCl_2_. The samples were heated at 95°C for 3 min, snap-cooled on ice for 10 min and incubated at room temperature for 2 h. The complexes were separated by electrophoresis through a 5% acrylamide/bisacrylamide (19:1) gel containing 0.1 mM MgCl_2_, during 45 min at 120 V. TBM buffer (45 mM Tris- Borate pH 8.5; 0.1mM MgCl_2_) was used as running buffer. The gels were then dried and subjected to autoradiography.

Additional methods are provided in Supplemental Information.

## RESULTS

### A *LEF1* NAT is differently expressed and spliced in epithelial and mesenchymal cells

**Figure 1.**
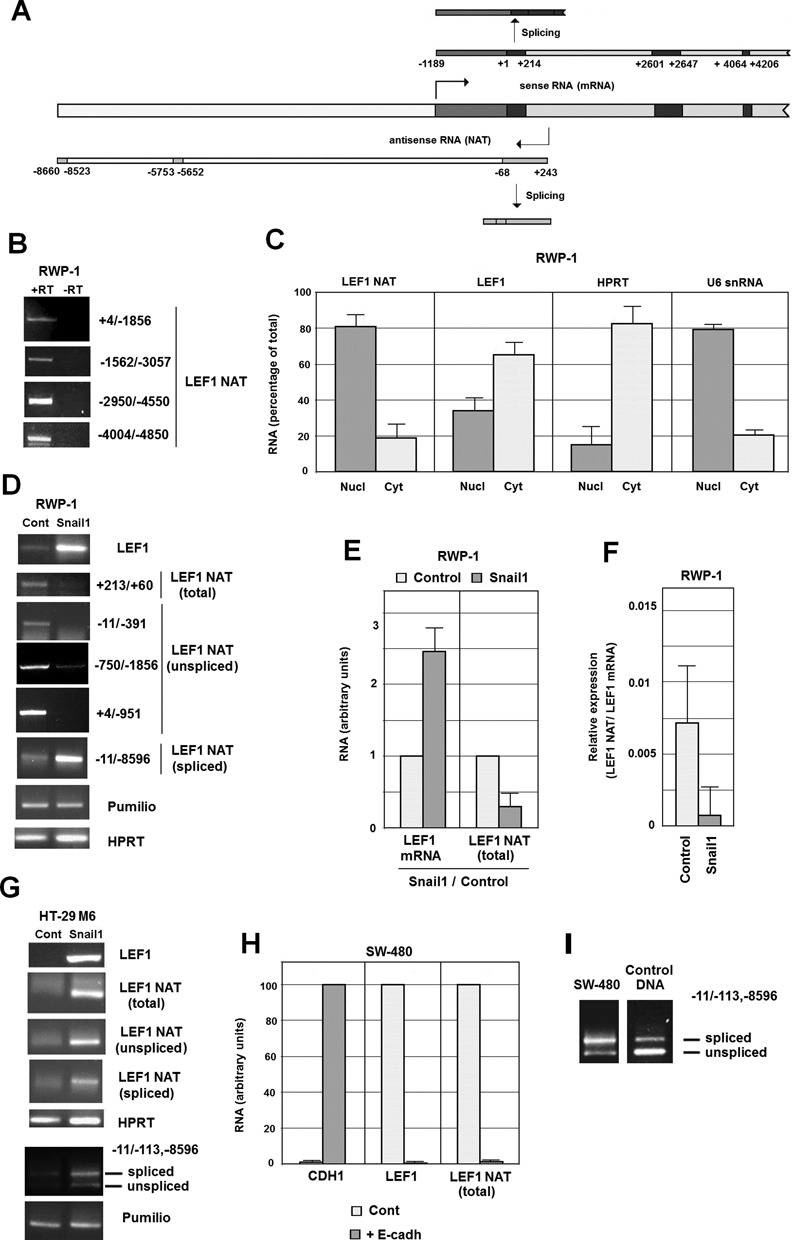
A *LEF1* natural antisense transcript (NAT) is expressed in the *LEF1* locus. **(A)** The diagram shows the sequence corresponding to the *LEF1* promoter (in white) and the *LEF1* mRNA. The 5′-UTR region is shaded in gray; the coding sequence, in dark gray and the processed introns, in light gray. The relative position of the NAT is also shown, indicating both the spliced and unspliced forms. The exons observed in the spliced NAT are indicated in light gray. +1 corresponds to the translation start site. **(B**–**I)** RNA was isolated from the indicated cell lines and analyzed by semi-quantitative (B, D, G, I) or quantitative (C, E, F, H) PCR after strand-specific retrotranscription (RT). In B and D, the oligos used for the RT-PCR are shown. C, cytosolic or nuclear RNA was obtained as indicated in Methods and the levels of total *LEF1* NAT (+213/+60), *LEF1* mRNA, *HPRT* (cytoplasmic control) or U6 snRNA (nuclear control) were determined by qRT-PCR. In, C, E, F and H, the values corresponded to the average ± SD of three-four experiments performed; in H, the error bars were lower than 3%. E and F, the relative abundance of total NAT with respect to *LEF1* mRNA was determined using strand-specific RT-PCR with +213 or +60 oligonuclotide and qPCR with the same pair of oligo nucleotides. The relative values were estimated in control or Snail1-transfected RWP-1 cells and represented as fold with respect to the value obtained in control cells (E) or relative NAT expression with respect to *LEF1* mRNA (F). G and I, the relative abundance of both NAT forms was assessed using specific RT with oligonucleotides starting at −8596 and −113, and semi-quantitative PCR, with these two oligos and another one, common for both spliced and unspliced NAT, starting at −11. Equimolar concentrations of two plasmids encoding the unspliced and spliced form of the NAT were used as controls in the PCR.

Expression of LEF1 is up-regulated in mesenchymal cells with respect to epithelial cells; therefore, as previously reported (15,16), induction of EMT by Snail1 transfection greatly increases *LEF1* mRNA in the different cell lines studied. The existence of NAT transcripts associated to the *LEF1* gene has been reported in the *Ensembl* database (Supplementary Figure S1A). Only one of the seven *LEF1* antisense transcripts described covers the *LEF1* promoter; this NAT (*LEF1-AS-001*) putatively starts at nucleotide +243 with respect to the *LEF1* start codon (indicated as +1), overlaps the main transcription start site positioned at −1189 (Figure 1A) (14), and ends at −8660. Indeed, we detected the expression of the unspliced form of this NAT using strand-specific RT-PCR in RWP-1 cells, a cell line with an epithelial morphology but also expressing low levels of mesenchymal markers, such as LEF1 (18). We verified that the start site of this NAT corresponded to nucleotide +243 using rapid amplification of 5′-cDNA ends (RACE). This *LEF1-AS-001* transcript, hereby named *LEF1* NAT, extends until −8660 since it was amplified by different oligonucleotide pairs specific for this transcript and not amplifying other *LEF1* antisense RNAs (Figure 1B), all with transcription start sites between −4025 and −5653 (Supplementary Figure S1A). Antisense transcription beyond −8660, presumably corresponding to AS-005 and AS-006, was also detected (Supplementary Figure S1B). The *LEF1* NAT was predominantly present in the nucleus in contrast to *LEF1* mRNA (Figure 1C). Using a quantitative RT-PCR (qRT-PCR) to determine NAT copy number in comparison to NAT +213/−1856 synthesized *in vitro* (see Supplemental Methods) we calculated that *LEF1* NAT was expressed at approximately 150–300 molecules per cell.

A spliced form of the NAT was also detected. We amplified a NAT band of 252 bp using oligonucleotides corresponding to −11 and −8596 (Figure 1D); sequencing of this band revealed that besides a fragment of the first exon (−11/−66) it contains only two other exons at −5753/−5652 and –8523/−8596, therefore, it corresponds to a variant of the reported spliced LEF1-AS001 transcript (ENST00000436413) that also contains sequences between −8024 and −8523 (Supplementary Figure S1A).

The relative expression of the two NAT variants (spliced or unspliced) was altered during EMT. As shown in Figure 1D, ectopic expression of Snail1 in RWP-1 up-regulated LEF1 and concomitantly decreased the total NAT levels, detected with a pair of oligonucleotides amplifying both forms (+213/+60). However, the two NATs were differently regulated since whereas the unspliced NAT was down-regulated, the spliced form was increased upon Snail1 expression.

We also sought to identify the elements controlling the expression of the *LEF1* NAT. Seeking to identify the NAT promoter, a DNA fragment corresponding to +66 to +857 was placed in the reverse orientation in the pGL3 plasmid (+857/+66, that corresponds to −614/+177 if we refer it to the transcription start site of the *LEF1* NAT). This DNA fragment, mainly corresponding to the first intron of *LEF1* mRNA (see Figure 1A), induced the expression of the reporter gene, with a higher activity in control RWP-1 cells than in cells expressing Snail1 (Supplementary Figure S2), correlating with the total levels of the NAT.

The relative expression of the LEF1 mRNA and NAT was also determined by strand-specific amplification and quantitative PCR with a common pair of oligonucleotides corresponding to a sequence present both in *LEF1* mRNA and NAT (+60/+213). We found that Snail1 expression increased *LEF1* mRNA almost 2.5-fold whereas it down-regulated total NAT by 65% (Figure 1E). Assuming that the priming by the reverse transcriptase was similar in both cases, we calculated that *LEF1* mRNA was much more abundant than the NAT in both cell lines: between 140-fold in control RWP-1 and almost 1000-fold in RWP-1 Snail1 cells (Figure 1F).

The differential expression of the spliced and unspliced forms of NAT was also determined in another model of EMT, HT-29 M6 cells control or transfected with Snail1. HT-29 M6 cells present a compact epithelial phenotype with high levels of E-cadherin and very low expression of mesenchymal genes; upon Snail1 expression cells undergo a complete EMT (19). As shown in Figure 1G, *LEF1* mRNA was not detected in control HT-29 M6 cells and was strongly induced by ectopic Snail1 expression. No expression of the NAT was observed in HT-29 M6 epithelial cells whereas both the spliced and unspliced forms were present in HT-29 M6 Snail1 mesenchymal cells (Figure 1G).

We also determined the relative abundance of the two different NAT forms in HT-29 M6 Snail1 cells by RT-PCR, in this case using a common oligonucleotide for both and two other specific for the spliced or unspliced NATs. As shown in Figure 1G, although the amplification of the spliced form was less efficient (see Figure 1I), this band was preferentially detected indicating that the spliced NAT was more abundant than the unspliced form in Snail1 expressing cells.

NAT levels were also determined in SW-480 cells. At low confluence, these cells express mesenchymal markers and low E-cadherin (20); ectopic E-cadherin transfection leads to a complete inhibition of the expression of *LEF1* and other mesenchymal genes (16, see also Figure 1H); thus, to a mesenchymal to epithelial conversion. We found that silencing of the *LEF1* locus was associated with a down-regulation of *LEF1* NAT (Figure 1H). Analysis of the relative abundance of the two NAT forms showed that, as happened in HT-29 M6 Snail1 cells, the spliced form is expressed at higher levels than the unspliced NAT in SW-480 cells (Figure 1I).

### Unspliced LEF1 NAT inhibits LEF1 transcription

**Figure 2.**
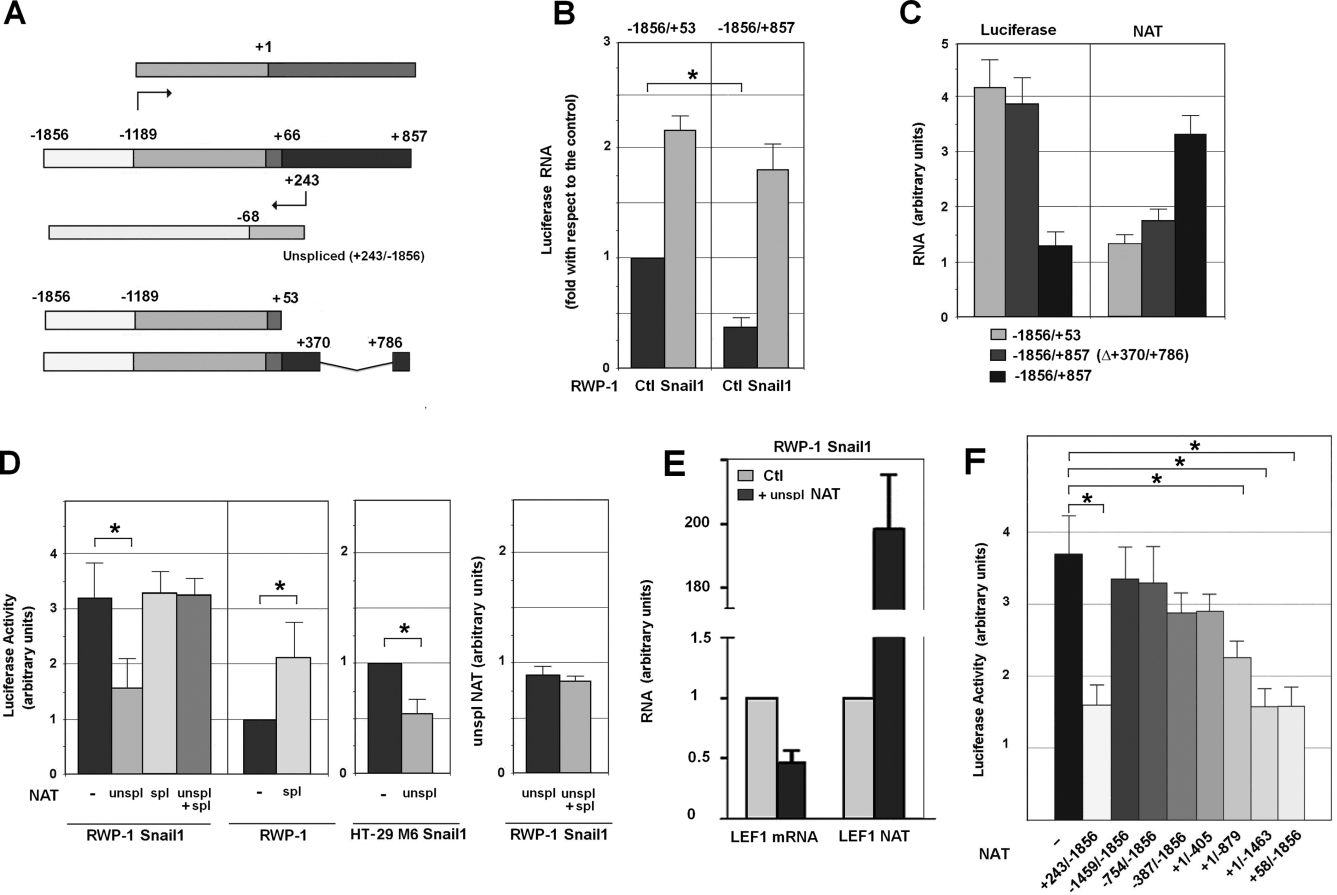
Unspliced *LEF1* NAT inhibits *LEF1* transcription. **(A)** A diagram of the *LEF1* gene, depicting the relative position of the NAT with respect to the main transcription start site (−1189) of the *LEF1* mRNA. The region −1856/−1189 correspond to the *LEF1* promoter. In dark gray, the first *LEF1* intron that contains the NAT promoter and NAT transcription start site, placed at +243. The diagram also shows the 1856/+53 DNA fragment containing the *LEF1* promoter but not the NAT promoter, and the −1856/+857 (Δ+370/+786) fragment, lacking the +370/+786 fragment of the NAT promoter but retaining the NAT transcription start site at +243. **(B)** RWP-1 and RWP-1 Snail1 cells were transfected with −1856/+857 or −1856/+53 *LEF1* gene fragments inserted in pGL3 vector and Renilla Luciferase (Luc) as control; the expression of Firefly and Renilla Luc were determined by qRT-PCR. Data are represented with respect to the level of expression of Firefly Luc under the control of −1856/+53 promoter in RWP-1 control cells**. (C)** RWP-1 cells were transfected with the constructs indicated below and the Renilla Luciferase, and the relative levels of Firefly Luciferase (Luciferase) or the NAT were determined by RT-PCR and referred to the levels of Renilla Luciferase. The figure shows the average ± SD of three experiments performed in duplicate. **(D, F)** Activity of the *LEF1* promoter was analyzed in the indicated cells transiently transfected with the different forms of the NAT in pcDNA3 plasmid and the pGL3-LEF1 promoter. The Luciferase activity values were calculated relative to the value from control HT-29 M6 or RWP-1 cells. The average ± SD of three experiments performed in triplicate is shown. An asterisk indicates that the differences are significant with a *P* < 0.05. In D, the relative expression of ectopically-expressed unspliced NAT in the presence or absence of co-transfected spliced NAT was determined as above. **(E)** RWP1 Snail1 cells, transfected with pBabe LEF1 NAT (+58/−1856) or empty pBabe, were subjected to nuclear run-on assay. The newly transcribed RNAs incorporating biotinylated UTP were isolated with magnetic streptavidin beads and quantified by RT-qPCR. The relative amounts of *LEF1* mRNA and *LEF1* NAT with respect to cell transfected with the control plasmid are shown. Pumilio was used as an internal control to normalize the transcription rates. The average ± SD of two independent experiments is presented.

As a first step to test for a potential role of the NAT in *LEF1* mRNA expression we analyzed the activity of a fragment of the *LEF1* promoter (−1856/+53) (Figure 2A) in RWP-1 control and Snail1 cells. As seen in Figure 2B, we found that the activity of this promoter was higher in mesenchymal than in epithelial cells, reflecting the expression of *LEF1* mRNA. The same experiments were repeated with another construction (−1856/+857) that contains both the *LEF1* mRNA and NAT promoters (Figure 2A). Instead of Luciferase activity, RNA expression of this gene was measured to eliminate the effects of different length in the 5′ UTR on Luciferase translation. Inclusion of the +53/+857 fragment significantly reduced the activity of the *LEF1* promoter in RWP-1 control cells to a greater extent than in Snail1 cells (Figure 2B), correlating with the activity of the NAT promoter (Supplementary Figure S2). As a consequence, the activity of the −1856/+857 promoter driving the expression of Luciferase was stimulated to a higher extent by Snail1 expression than that of the −1856/+53 promoter.

We validate these results using another DNA construct, −1856/+857 (Δ+370/+786) in which a considerable part of the NAT promoter was deleted although the NAT transcription start site remained (Figure 2A). We determined Luciferase RNA and NAT expression relative to the endogenous levels in these cells. Again, the decrease in Luciferase RNA, corresponding to the activity of the *LEF1* promoter, correlated with the levels of NAT expression from the construct. Therefore, these experiments are consistent with the luciferase expression driven by the *LEF1* promoter being repressed by the NAT acting in *cis*.

We also determined if NAT inhibition of the *LEF1* promoter activity could also be observed in *trans* by analyzing the effect of a transfected NAT-expressing plasmid on *LEF1* promoter activity. A +243/−1856 NAT, a fragment unable to be spliced because it lacks the 3′-acceptor site, inhibited the *LEF1* promoter (determined by Luciferase activity) in RWP-1 and HT-29 M6 Snail1-expressing cells (Figure 2D). Unspliced *LEF1* NAT also decreased transcription of *LEF1* mRNA as determined by nuclear run-on assays (Figure 2E).

Contrary to the unspliced NAT, the spliced transcript did not repress the *LEF1* promoter and even prevented the inhibition caused by the unspliced NAT in RWP-1 Snail1 cells (Figure 2D, left panel) without altering the levels of this form (Figure 2D, right panel). This result indicates that the spliced NAT acts as a natural dominant negative form of the unspliced NAT. Indeed, the spliced NAT up-regulated *LEF1* promoter activity in RWP-1 cells (Figure 2D).

We also analyzed the activity of different unspliced NAT deletion mutants. As shown in Figure 2F, both the +58/−1856 and +1/−1463 NATs inhibited *LEF1* promoter activity to a similar extent as the full-length NAT (+243/−1856). A +1/−879 NAT sequence significantly repressed the promoter whereas shorter fragments, with elimination of 5′ or 3′ sequences, were not active (Figure 2F). Therefore, the +1/−1463 NAT, overlapping the 5′-UTR and the *LEF1* proximal promoter, contained all the elements required for *LEF1* promoter inhibition.

**Figure 3.**
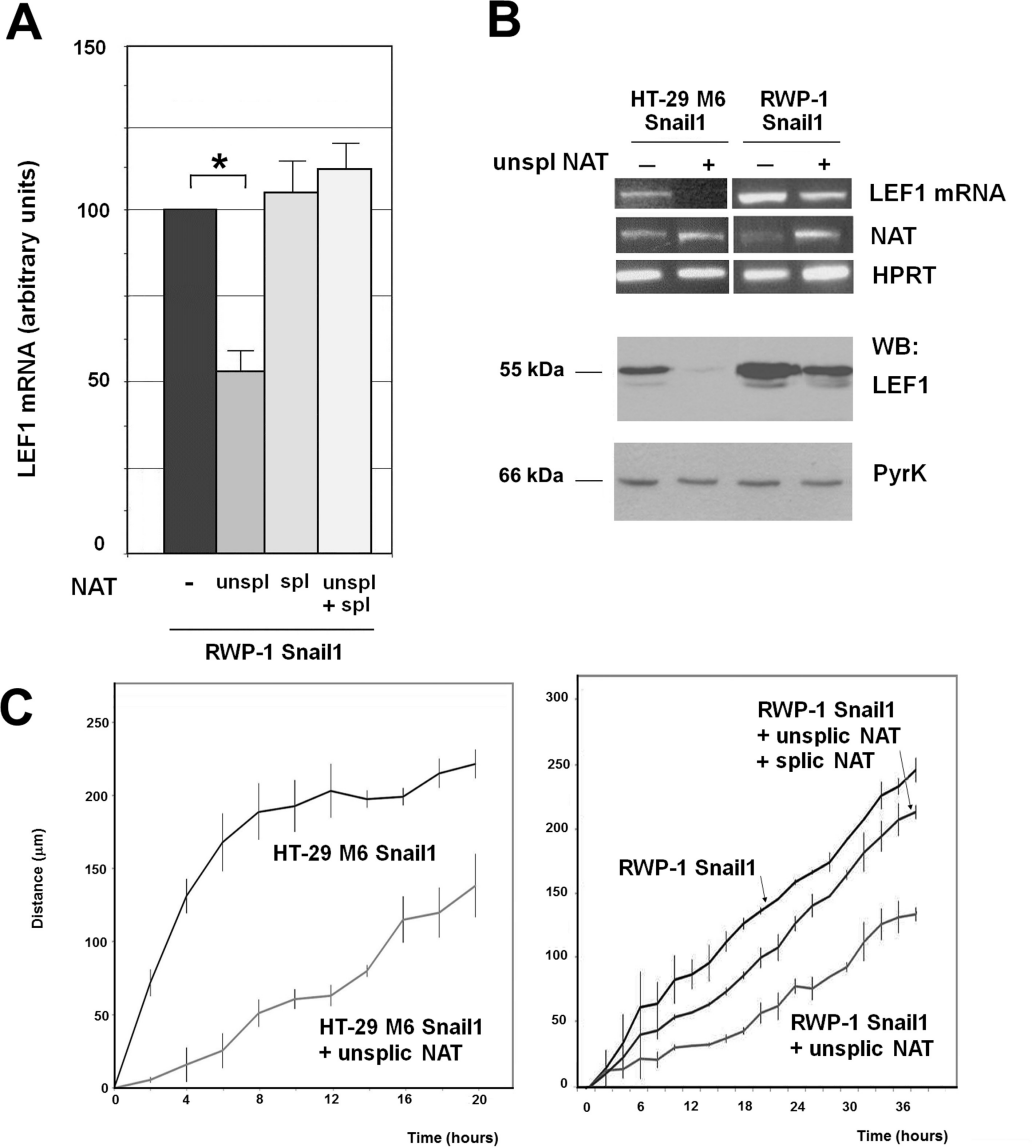
*LEF1* NAT controls the expression of *LEF1* mRNA and protein. **(A)** Unspliced (+213/−1856) or spliced NAT were synthesized *in vitro*. A three-fold excess spliced NAT with respect to the unspliced form were transfected in the conditions indicated in Methods. In the control samples an irrelevant RNA (corresponding to a fragment of pcDNA3 plasmid) was transfected. After 36 h, RNA was obtained and levels of LEF1 mRNA were determined using two oligonucleotides corresponding to an amplicon present in the third exon. The results correspond to the average ± range of two experiments performed in duplicate. The asterisk indicates significant (*P* < 0.05). **(B)** RWP-1 or HT-29 M6 Snail1 cells were stably transfected with pBabe-LEF1 NAT (unspliced) or pBabe as control. RNA was collected and analyzed by RT-PCR with oligonucleotides specific for *LEF1* mRNA, *LEF1* NAT (total) or *HPRT* as control (top panel); alternatively protein extracts were prepared and analyzed by western blot with a polyclonal antibody against LEF1 (Cell Signal) or anti PyrK (Sigma) (bottom panel). **(C)** The migration capacity of the indicated cell populations was determined as described in Methods. The differences are significant with a *P* < 0.05.

The effect of the NAT on *LEF1* mRNA expression was also determined by transfecting the spliced and unspliced forms of the transcript. We performed this analysis by two alternative experimental approaches. First, we transfected *in vitro* synthesized RNA corresponding to these NATs and checked *LEF1* mRNA expression by strand-specific qRT-PCR. As shown in Figure 3A, the unspliced form down-regulated *LEF1* mRNA whereas the spliced NAT did not have a significant effect. Co-transfection of the spliced NAT inhibited the action of the unspliced form, suggesting that the spliced NAT is acting as a dominant negative factor (Figure 3A). We also stably transfected a plasmid expressing the unspliced form of the transcript. As shown in Figure 3B (top), the unspliced NAT down-regulated *LEF1* mRNA both in HT-29 M6 and RWP-1 Snail1 cells, without altering the stability of this RNA (see Supplementary Figure S3). A similar inhibition of LEF1 protein by unspliced NAT was also observed (Figure 3B bottom). Moreover, unspliced LEF1 NAT expression decreased the migration of both cell lines (Figure 3C) without affecting their growth rate (Supplementary Figure S4). Co-transfection of spliced NAT remarkably rescued the inhibition of migration caused by unpsliced NAT in RWP-1 Snail1 cells (Figure 3C). We did not detect a significant change in the phenotype of these cells. LEF1 NAT only caused a small increase in the mRNA levels of E-cadherin and other epithelial genes (Claudin4, Occludin) (Supplementary Figure S5A); E-cadherin up-regulation was also detected at protein level (Supplementary Figure S5B). No changes in the E-cadherin repressors Snail1 or Zeb1 (2,3) were observed, however *ZEB2* RNA was significantly down-regulated (Supplementary Figure S5A), suggesting that LEF1 was contributing to the expression of this gene.

### *LEF1* NAT binds to the *LEF1* promoter

**Figure 4.**
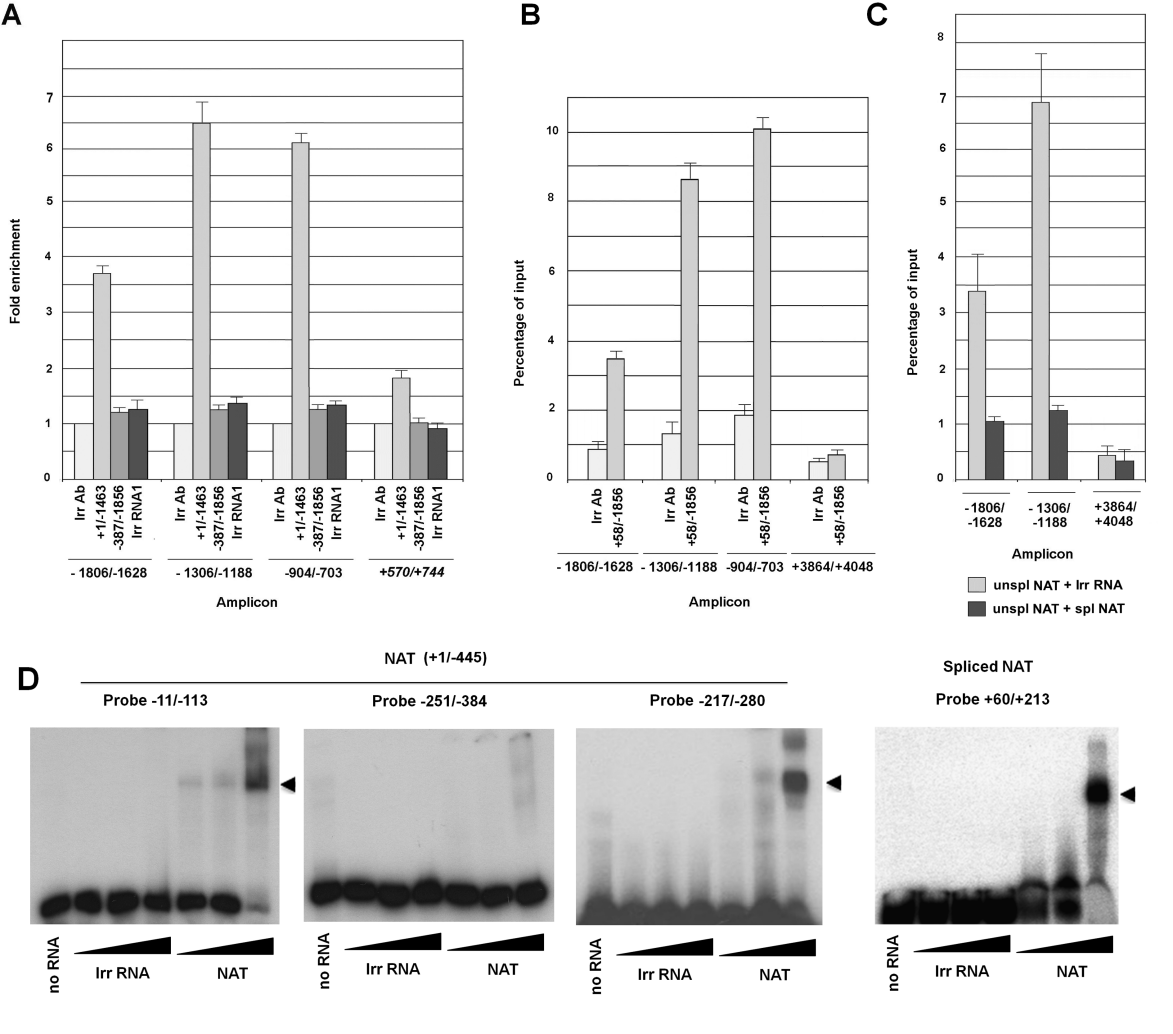
*LEF1* NAT binds to the *LEF1* promoter. **(A)** RWP-1 cells were transfected with the pGL3-LEF1 promoter (−1856/+58) plasmid and with either *in vitro* synthesized biotinylated unprocessed NAT or an irrelevant RNAs encoding Cre (Irr RNA). After 24 h, cells were fixed with formaldehyde as described in Methods. Extracts were incubated with an anti-biotin antibody and immunoprecipitated with protein A-agarose. Presence of the indicated amplicons was assessed by qPCR as described in the Methods. (**B, C**) NAT binding to endogenous *LEF1* promoter was analyzed as in (A) but without transfection of pGL3 LEF1 promoter. In C, similar assays were performed transfecting also *in vitro* synthesized (not-biotinylated) spliced NAT or an irrelevant RNA. The results represent the average ± SD of three experiments. (**D**) Binding of the +1/−445 fragment of unspliced NAT or of the spliced NAT to ^32^P-labeled DNA probes was measured by EMSA as indicated in the Methods. The migration of the shifted band (potentially triplex DNA-RNA) is indicated by a closed arrow head.

We considered that the repression of the *LEF1* promoter by the NAT may involve an interaction of the RNA with this DNA element. To test this, biotinylated NAT (+1/−1463) and pGL3-LEF1 promoter were transfected into RWP-1 cells which were then crosslinked with formaldehyde and the NAT was precipitated from total extracts using an anti-biotin antibody. As shown in Figure 4A and Supplementary Figure S6, sequences corresponding to the proximal *LEF1* promoter (−904/−703, −1306/−1188 and −1806/−1626) were enriched in these complexes, indicating that NAT is associated with these elements. Very little interaction was detected with another amplicon corresponding to luciferase (+570/+744) or to another different co-transfected promoter, CDH1 (Supplementary Figure S6). The specificity of the NAT-promoter interaction was further demonstrated by the absence of binding detected with two irrelevant RNAs (Figure 4A and Supplementary Figure S6). A NAT fragment lacking the 5′ end (-387/−1856), and unable to repress the *LEF1* promoter (Figure 2F), did not bind to the *LEF1* promoter, suggesting that the association requires the +1/−387 sequence (Figure 4A). Binding of biotinylated NAT to the endogenous LEF1 promoter was also detected (Figure 4B); in these experiments an amplicon corresponding to the second intron of the *LEF1* gene was used as a negative control (Figure 4B and C). NAT binding to endogenous *LEF1* promoter was abrogated if the spliced NAT was co-transfected with the unspliced form of the NAT (Figure 4C).

We also determined if binding of the NAT to the DNA could also be detected *in vitro*. In these experiments, we used a fragment of the NAT (+1/−445) corresponding to the sequence required *in vivo* for the binding to *LEF1* promoter. As shown in Figure 4D, this RNA induced the formation of a high molecular weight triplex structure when a ^32^P-labeled −11/−113 DNA probe was used. As expected, an irrelevant RNA did not retard the migration of this probe. The generation of the triplex DNA-RNA was sequence-specific since it was detected with a −217/−280 DNA but not with a −251/−384 fragment (Figure 4D). The spliced NAT, comprising +243/−68 and downstream sequences (−5753/−5651 and −8523/−8596) also caused a shift in the mobility of a +60/+213 probe indicating that it retains the capability to interact with the DNA (Figure 4D).

### *LEF1* NAT targets PRC2 complex to *LEF1* promoter

**Figure 5.**
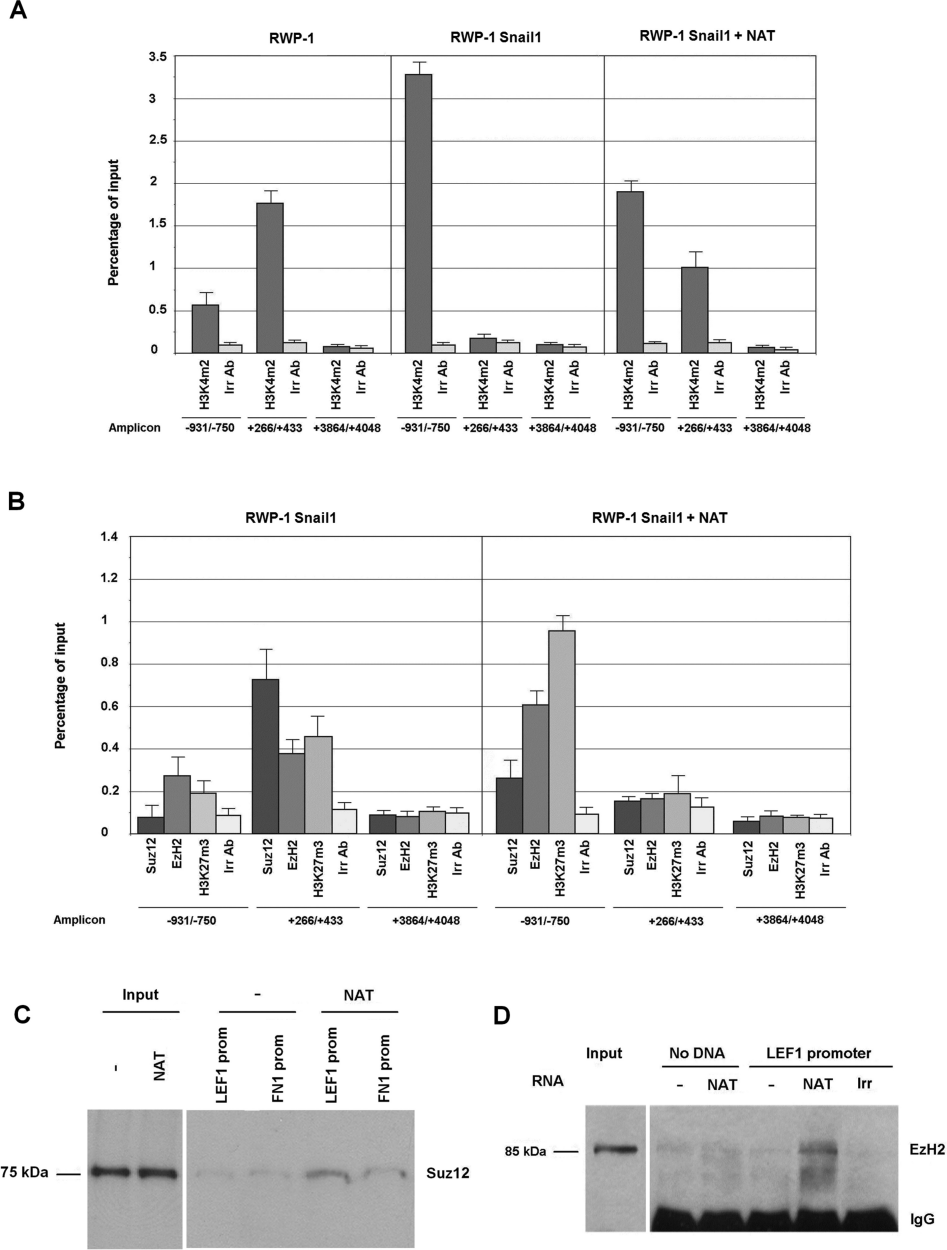
*LEF1* NAT recruits PRC2 to the *LEF1* promoter. **(A, B)** ChIP assays were performed in RWP-1, RWP-1 Snail1 or RWP-1 Snail1 cells transfected with unspliced *LEF1* NAT. The immunoprecipitations were performed with the indicated antibodies or with an irrelevant IgG control. The presence of amplicons corresponding to the *LEF1* promoter (−931/−750), the NAT promoter (+266/+433) or an irrelevant DNA sequence (+3864/+4068) was determined. The association to these sequences with H3K4me2, an epigenetic mark characteristic of active promoters, is shown in panel A and with H3K27me3, a mark associated to inactive promoters, or of the two members of PRC2 complex, which sets this mark, in panel B. The average ± SD of three experiments performed in triplicate is shown. **(C, D)** BOPA assays were performed with the biotinylated −1856/+58 *LEF1* promoter and cell extracts from RWP-1 Snail1 transfected or not with unspliced NAT (C). In D, when indicated, *in vitro* synthesized NAT or an irrelevant RNA (YB1X) were added. Samples were incubated with an anti-biotin antibody, the oligonucleotide was pulled-down with protein G-agarose, and the presence of Ezh2 in the precipitate was analyzed by western blot.

We hypothesized that repression of *LEF1* transcription by *LEF1* NAT was also associated with a switch in the histone modification marks at the *LEF1* promoter. To test this, we determined the presence of two marks at the LEF1 promoter, dimethylation at Lys4 of histone 3 (H3K4me2) and trimethylation of Lys27 in Histone 3 (H3K27me3), which are associated with promoter activation or repression, respectively (21). In these assays we amplified sequences corresponding to the *LEF1* promoter (−931/−750 amplicon), the NAT promoter (+266/+435 amplicon), or a control DNA sequence (+3864/+4048). As seen in Figure 5A, the H3K4me2 mark was present at the *LEF1* promoter to a much greater extent in RWP-1 Snail1 cells than in control RWP-1 cells. Conversely, H3K4me2 was detected at the NAT promoter to a greater degree in RWP-1 than in RWP-1 Snail1 cells, correlating with the higher expression of the NAT in these cells. Ectopic transfection of the NAT in RWP-1 Snail1 cells reverted this pattern to one similar to that observed in RWP-1 cells; therefore, the presence of the NAT down-regulated H3K4me2 at the *LEF1* promoter and up-regulated it at the NAT promoter.

The opposite results were obtained for H3K27me3. NAT over-expression increased this repressive mark at the *LEF1* promoter and decreased it at the NAT promoter (Figure 5B). Since methylation at Lys27 is a consequence of the catalytic activity of Polycomb Repressive Complex 2 (PRC2) (22), we investigated the binding of subunits of this complex to these two promoters. Two core subunits of this complex, Suz12 and Ezh2, displayed a binding pattern similar to the pattern obtained for the H3K27me3 mark (Figure 5B). Therefore, expression of the NAT induced the association of Suz12 and Ezh2 with the *LEF1* promoter.

We also verified whether the presence of the NAT promotes PRC2 binding to the *LEF1* promoter using two *in vitro* assays. For this, we synthesized a biotinylated DNA corresponding to the *LEF1* promoter (−1856/+58). A cell extract, prepared in RNAse-free conditions from RWP-1 Snail1 cells transfected with a control or a NAT-expressing plasmid, was incubated with the biotinylated *LEF1* promoter or a fragment of the FN1 promoter of the same size as control. As shown in Figure 5C, binding of the PRC2 component Suz12 was only detected at the *LEF1* promoter and only when NAT-expressing cells were used. We also performed the alternative experiment, in which a cell extract from RWP-1 Snail1 cells was incubated with biotinylated LEF1 promoter in the presence of *in vitro* transcribed NAT or an irrelevant RNA. As show in Figure 5D, the PRC2 component Ezh2 interacted with the LEF1 promoter only when the NAT was present, indicating that the NAT facilitates the recruitment of PRC2 to this promoter.

**Figure 6.**
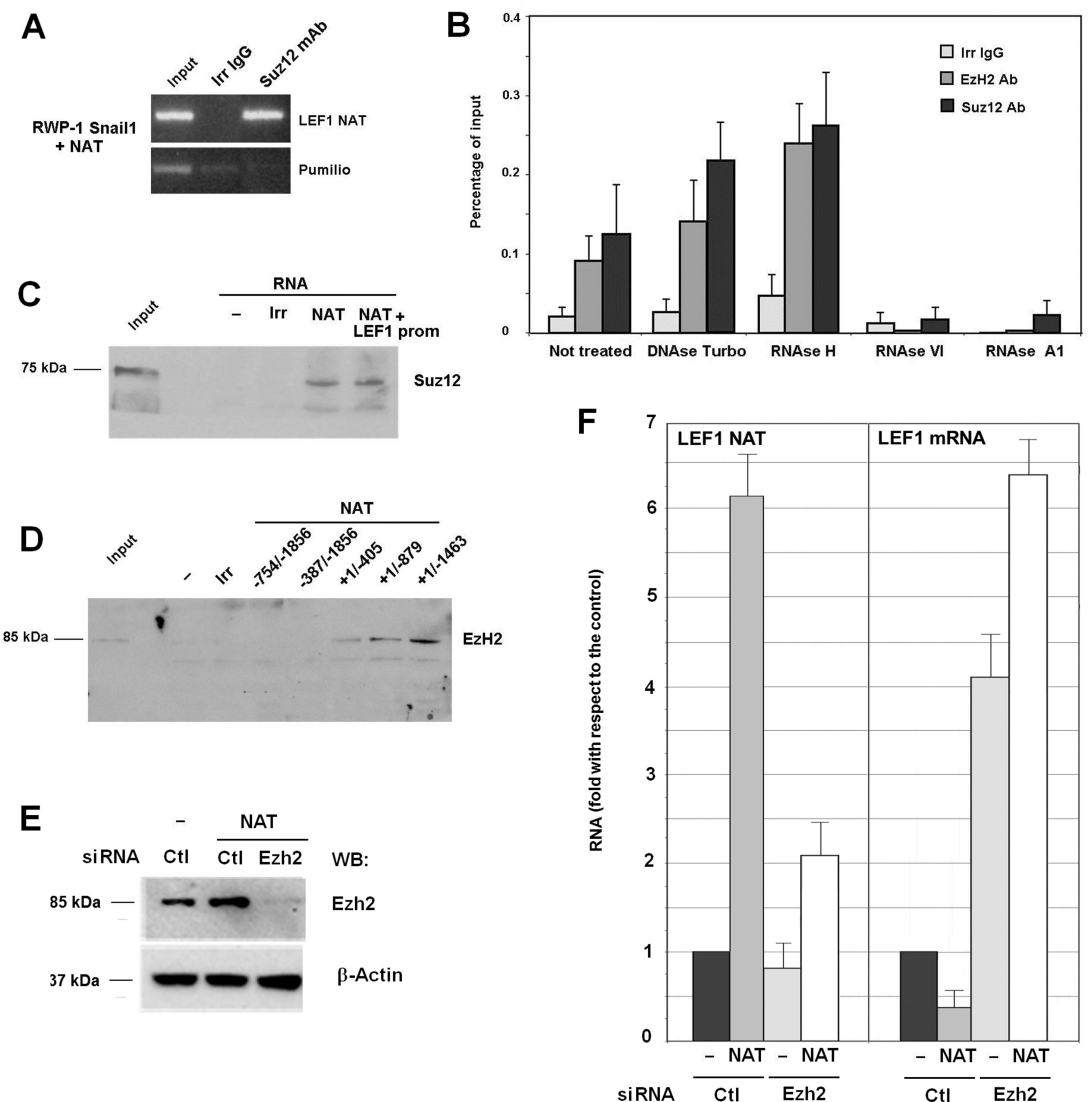
*LEF1* NAT directs PRC2 binding to *LEF1* promoter. **(A)** RIP assays were performed in RWP-1 Snail1 NAT cells with an antibody against Suz12 or and irrelevant IgG, and presence of *LEF1* NAT was analyzed by semi-quantitative RT-PCR. **(B)** RIP assays were also performed in RWP-1 cells with Ezh2 or Suz12 antibodies and analyzed by qRT-PCR. Cell extracts were treated with the indicated nucleases prior to immunoprecipitation. The mean ±SD of three experiments performed in triplicate is shown. **(C, D)** RNA-BOPA assays were performed using 6 μg of biotinylated NAT and total cellular extracts from RWP-1 cells. The presence of the PRC2 subunits Ezh2 and Suz12 was determined by western blot. (**E, F**) RWP-1 Snail1 cell stably expressing the unspliced NAT when indicated were transfected with siRNA specific for Ezh2 or a scrambled siRNA as control. (E), the extent of Ezh2 down-regulation was determined by western blot. (F), the levels of *LEF1* NAT and mRNA were determined by qRT-PCR as above and calculated relative the value obtained in cells without NAT expression.

Several studies have demonstrated that lncRNAs are physically associated with PRC2 (23,24). Therefore, we determined whether PRC2 interacted with LEF1 NAT using RNA immunoprecipitation (RIP) coupled to RT-PCR. NAT was effectively co-immunoprecipitated by an antibody against Suz12 but not by an irrelevant control antibody (Figure 6A). The assay was also carried out using quantitative PCR and pretreating the immunocomplexes with different nucleases. Incubation with ribonucleases (RNases) that digest single-stranded RNA (RNase A1) or double-stranded RNA (RNase VI) abolished RIP signals, whereas treatments with RNase H (which cleaves RNA in RNA:DNA heteroduplexes) and DNase I did not prevent the immunoprecipitation of NAT with the Suz12 or the Ezh2 antibody (Figure 6B). Therefore, the PRC2 complex interacts with the NAT RNA and not with the RNA/DNA complexes.

Biotinylated-oligonucleotide pull-down (BOPA) assays were also performed using a biotinylated NAT. The PRC2 components Suz12 and Ezh2 were copurified with this NAT when this transcript was incubated with a cell nuclear extract (Figure 6C, D). As a control, lower interaction was observed between the PRC2 complex and an irrelevant biotinylated RNA. The presence of the *LEF1* promoter did not increase Suz12-NAT binding, supporting our finding that DNA is not required for this association.

We mapped the elements in *LEF1* NAT required for PRC2 binding. The NAT +243/+1 sequence was not required for the inhibition of *LEF1* promoter, suggesting that this segment did not contain the binding element. RNA-BOPA assays confirmed this conclusion since Ezh2 co-precipitated with the +1/−1463 fragment of NAT (Figure 6D). Progressive deletion of elements downstream in the NAT did however affect the interaction; Ezh2 bound more efficiently to +1/−1463 NAT than to +1/−879 and +1/−405 fragments. No association was observed between PRC2 and −754/−1856 or −387/−1856 sequences. Therefore, we conclude that binding requires an element located between +1 and −405, although other sequences situated downstream are also necessary.

We also broadened these studies beyond PRC2 and found that other proteins were enriched in NAT-bound complexes compared with an irrelevant control RNA (Supplementary Figure S7). They correspond to RNA-binding proteins without any known role in EMT. Their involvement in NAT processing and *LEF1* mRNA inhibition remains to be established.

Finally we also sought to determine the relevance of Ezh2 for NAT function. We depleted Ezh2 protein in RWP-1 Snail1 cells using a specific siRNA (25) (Figure 6E) and analyzed the effect of transfected NAT in these cells. As above, NAT transfection down-regulated *LEF1* mRNA in cells transfected with a scrambled siRNA control (Figure 6F). NAT did not decrease the expression of this gene in Ezh2-depleted cells; however, this result was not conclusive since the levels of the ectopic transcript were remarkable decreased by Ezh2 down-regulation (Figure 6F). These results suggest that binding to Ezh2 is required for NAT stability and further support a functional relationship between the NAT and PRC2 complex.

## DISCUSSION

In recent years the function of long non-coding RNAs (lncRNAs) has started to be unveiled. The broad functional repertoire of these RNAs includes roles in high-order chromosomal dynamics and subcellular structural organization (26,27). One major theme emerging is the involvement of these ncRNAs in regulating the transcription of neighboring protein-coding genes (28). In this article we have characterized a natural antisense transcript that modulates the expression of the mesenchymal-specific gene *LEF1*. This NAT is expressed using a promoter located in the first intron of the *LEF1* gene, a feature common to many NATs. Localized in the nucleus, this NAT overlaps the 5′ UTR region and the promoter of the *LEF1* gene. Moreover, it binds to and inhibits the proximal *LEF1* promoter, suggesting that it acts *in cis* whereas it is being synthesized. This NAT can undergoes splicing, generating a shorter antisense transcript, only 300 b in length and encompassing sequences close to the *LEF1* start codon. The spliced NAT also binds to these elements and prevents the interaction of the unspliced form with *LEF1* promoter, precluding the inhibition caused by this transcript. Therefore, spliced NAT works as a natural dominant negative inhibitor of the unprocessed form, mainly in mesenchymal cells.

**Figure 7.**
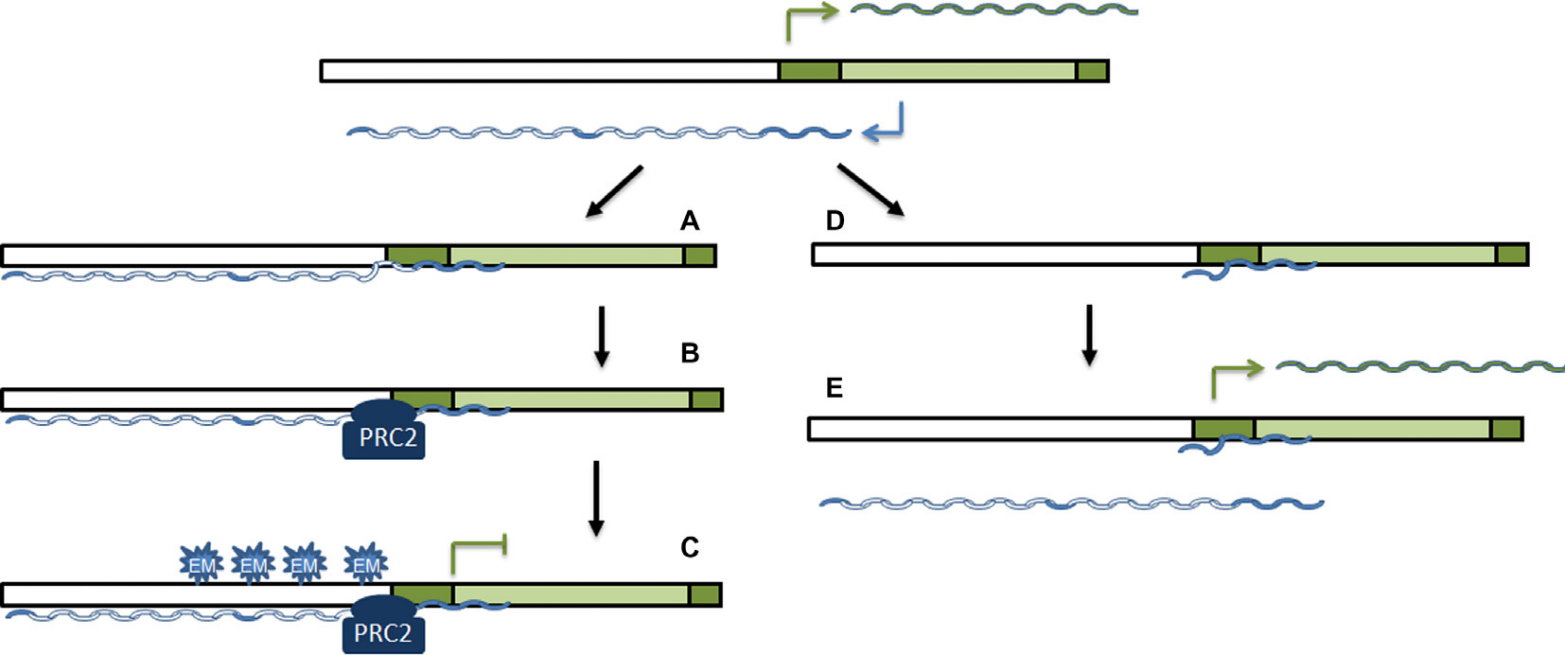
A model for the regulation of *LEF1* mRNA expression by *LEF1* NAT. Contrarily to epithelial cells, where the gene is silenced and neither the *LEF1* promoter nor the NAT promoter are active, during EMT both promoters are triggered to produce *LEF1* mRNA (in green) and NAT (in blue) transcripts. In cells with an intermediate or epithelial metastable phenotype, NAT is not processed and retains the long intron (in white). Mainly through the formation of triplex RNA-DNA structures with the proximal promoter, the most 5′ sequences of the NAT interact with the DNA (**A**). Moreover, the NAT also associates with PRC2, an interaction that requires 5′ sequences and also likely dependent on the length of the RNA (**B**). Recruitment of PRC2 enables the deposition of the repressive mark H3K27me3 and the inhibition of *LEF1* promoter (**C**). In fully mesenchymal cells, although the promoter retains activity, the NAT is rapidly processed generating a much smaller transcript that, although capable of interacting with the *LEF1* promoter (**D**) is unable to bind the PRC2 complex and prevent *LEF1* RNA synthesis (**E**). Moreover, since it competes with the interaction of the remaining unspliced NAT, the spliced NAT prevents it from acting on *LEF1* promoter. In this figure the *LEF1* promoter is shown in white and the NAT promoter, contained in the sequence corresponded to the first *LEF1* mRNA intron, in light green.

The action of the two NAT forms on *LEF1* expression is tightly regulated. According to our interpretation of the results, in epithelial cells, such as HT-29 M6, this gene locus is silent and neither *LEF1* mRNA nor the NAT are transcribed. However, in cells with mesenchymal-epithelial intermediate features, expressing epithelial genes but also presenting low levels of mesenchymal markers, such as RWP-1 cells, both promoters are activated. In these cells, *LEF1* NAT is not substantially processed and interacts with the *LEF1* promoter (Figure 7a). Although the most 5′ sequences of the NAT are capable of interacting with the DNA, possibly through the formation of triplex DNA-RNA structures we cannot rule out that other more 3′ elements can also contribute to the binding. In addition, unpsliced NAT recruits PRC2 through direct interaction (Figure 7b) and attenuates the activity of this promoter by depositing H3K27me3 marks (Figure 7c). In mesenchymal cells the effect of the NAT on *LEF1* transcription is prevented by its splicing which generates a form that can also interact with the *LEF1* promoter (Figure 7d) but lacks the elements required for PRC2 binding, therefore being unable to prevent *LEF1* mRNA transcription (Figure 7e). Moreover, the spliced NAT competes with the action of the unspliced form. Consequently, the expression of *LEF1* is controlled through the activity of two alternative promoters and the processing of the transcripts that are generated. These results reinforce previous results demonstrating the regulation of gene expression by the activity of alternative promoters placed in the same gene (29,30).

Recent reports have demonstrated that non-coding RNAs can direct PRC2 binding to gene promoters. This is the case for HOTAIR (31,32), Xist (23), Kcnq1ot1 (33,34) and Gtl2 (24). Perhaps the most prominent example is silencing of the inactive X-chromosome by the ncRNA Xist. To normalize the copy number of X-chromosome between male and female cells, Xist RNA from one of the two female X-chromosome recruits PRC2 to trimethylate histone H3 at lysine 27 (H3K27me3), rendering the chromosome transcriptionally silent (33). More specifically, a 1.6 kb ncRNA (RepA) within Xist is responsible for the PRC2 interaction, with Ezh2 serving as the RNA-binding subunit (23). Another example is HOTAIR, a long intergenic RNA transcribed from the HOXC cluster that represses genes in the HOXD cluster by binding to PRC2 (32). Recent results indicate that HOTAIR also recruits PRC2 to many other genes, suggesting that it participates in the mechanism of repression by this complex (35). Moreover, HOTAIR also interacts with LSD1 (36), thus assembling PRC2 with the LSD1/CoREST/REST complex, and maybe with other proteins associated with LSD1, such as the Snail1 transcriptional repressor (37).

However, these results have been questioned. Cech et al have shown that the recombinant PRC2 complex has the same affinity for HOTAIR as for an irrelevant bacterial RNA (38). Binding to RNA is mainly dependent on RNA size, probably due to the higher capability to adopt secondary structures. These results have been recently refined by Lee et al who have demonstrated that other subunit of the complex Eed decrease the binding of the core PRC2 complex (Ezh2, Suz12) to RNA (39). Moreover, a recent report has shown that PRC2 binding to RNA depends on length but also of the intrinsic characteristics since size-matched RNAs present relevant differences in affinity for the complex (40). Our results fit well with this model. Although not totally dependent on length, since a −387/−1856 fragment did not show interaction whereas +1/−483 did, binding of *LEF1* NAT to PRC2 is sensitive to size and progressive 3′ deletions decrease the interaction. We concluded that although +1/−405 was required for binding, presence of additional sequences is also needed for a functional interaction. It is noteworthy that the +1/−405 fragment contains a much higher proportion of GC (75%) than the rest of the transcript suggesting that it might have a higher tendency to form stable secondary structures.

Non-specific PRC2 binding to RNA has been suggested to have a role in preventing inappropriate transcription from PRC2-targeted genes (38). Cryptic transcripts would recruit PRC2 to recognize previously deposited H3K27me3 and reinforce this mark. Our results suggest that this recruitment might also be (or mainly) mediated by antisense transcripts synthesized from a promoter present in the same gene. It is likely that, as happens in the case of *LEF1* NAT, the association of PRC2 would be prevented by splicing of the sense transcript, that markedly decrease transcript size, coupling the two processes of RNA transcription and splicing.

## SUPPLEMENTARY DATA

Supplementary Data are available at NAR Online.

SUPPLEMENTARY DATA
