# Correction to ‘Splicing of a non-coding antisense transcript controls LEF1 gene expression’

**DOI:** 10.1093/nar/gkag725

**Published:** 2026-07-14

This is a correction to: Manuel Beltran, Estel Aparicio-Prat, Rocco Mazzolini, Alba Millanes-Romero, Pere Massó, Richard G. Jenner, Víctor M. Díaz, Sandra Peiró, Antonio García de Herreros, Splicing of a non-coding antisense transcript controls LEF1 gene expression, Nucleic Acids Research, Volume 43, Issue 12, 13 July 2015, Pages 5785–5797, https://doi.org/10.1093/nar/gkv502

In December 2025, concerns were raised on PubPeer (https://pubpeer.com/publications/49B620CF0345268498C4059925E2E3#1) regarding a potential duplication of LEF1 mRNA bands in Figures 1B and 3B. Although there is not any band duplicated between these two figures, following a review of the original data the corresponding author wishes to provide a correction to Figure 3B.

The corresponding author confirms that the LEF1 RT–PCR image presented in Figure 3B was mislabelled. Specifically, the panel labelled as RWP1 Snail1 ± NAT actually represents HT–29 M6 Snail1 ± NAT, while the panel labelled HT–29 M6 Snail1 ± NAT corresponds to RWP1 Snail1 ± NAT. The corrected panel assignments are consistent with the original experimental data and with the results reported in the first author’s doctoral thesis. Both are included in the Supplementary Data accompanying this notice.

In addition, a lane of the same LEF1 RT–PCR experiment was used in both Figure 1G and Figure 3B. This shared lane in these figures corresponds to the same sample (see Supplementary data).

A corrected version of Figure 3B, with the panels properly labelled, is provided in this correction notice. The authors confirm that this labelling error does not affect the results or conclusions of the article, which are supported by additional experiments presented elsewhere in the study (including Figures 2 and 3A) and by previously published work.

**Figure:**
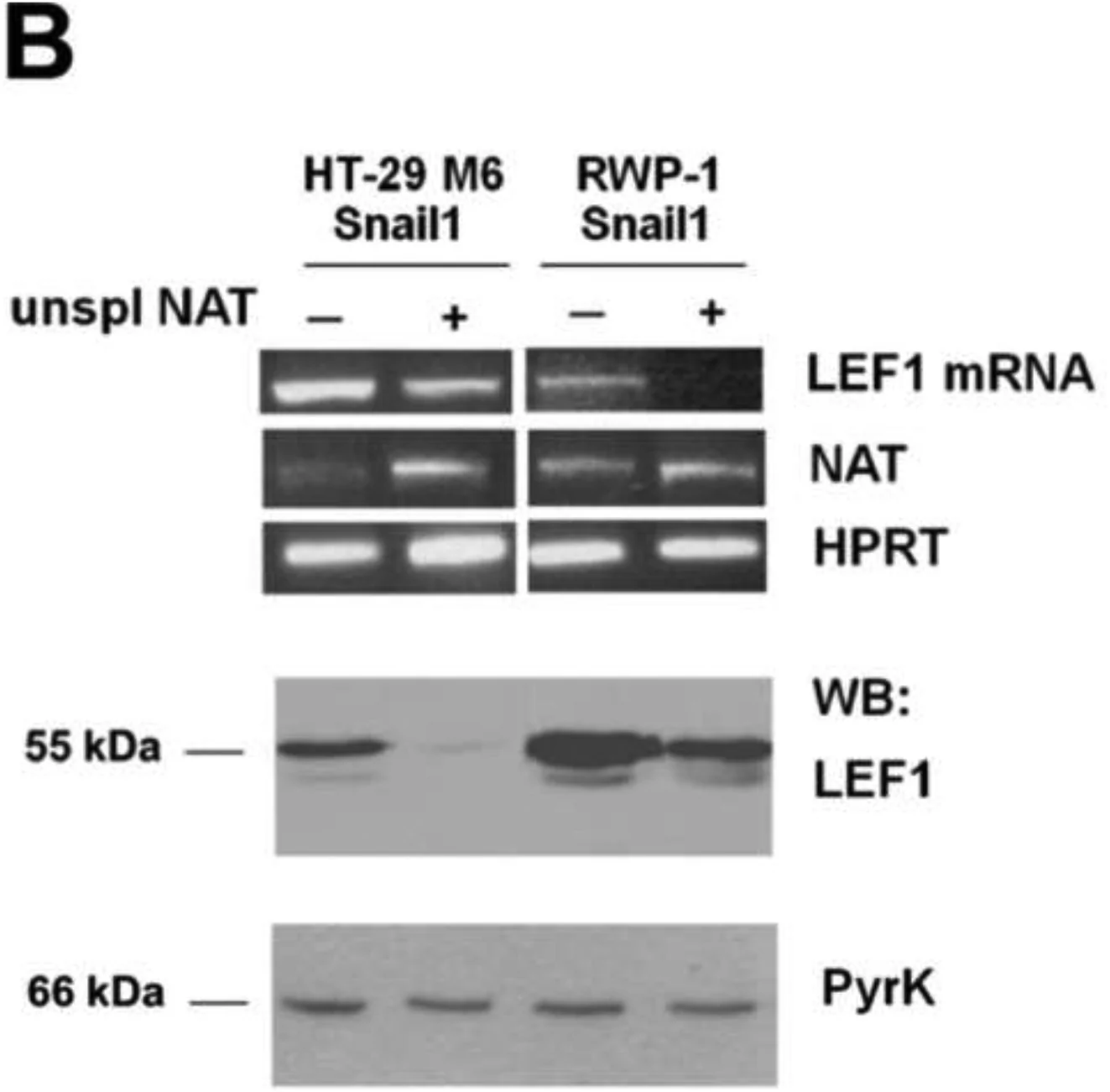


The authors sincerely apologize for the error and for any confusion it may have caused.

To preserve the published version of record, Figure 3B has been corrected only in this correction notice.

## Supplementary Material

gkag725_Supplemental_File

