# Supplementary material for: Splicing of a non-coding antisense transcript controls LEF1 gene expression
Source: Nucleic Acids Res. 2015 May 18;43(12):5785–97. doi: 10.1093/nar/gkv502 (PMC4499130; doi:10.1093/nar/gkv502)
Supplement: SUPPLEMENTARY DATA [file supp_43_12_5785__index.html]

Splicing of a non-coding antisense transcript controls LEF1 gene expression — Splicing of a non-coding antisense transcript controls LEF1 gene expression — SUPPLEMENTARY DATA 

# Splicing of a non-coding antisense transcript controls *LEF1* gene expression

## SUPPLEMENTARY DATA

- SUPPLEMENTARY DATA
