# Supplementary material for: Splicing of a non-coding antisense transcript controls LEF1 gene expression
Source: Nucleic Acids Res. 2015 May 18;43(12):5785–97. doi: 10.1093/nar/gkv502 (PMC4499130; doi:10.1093/nar/gkv502)
Supplement: SUPPLEMENTARY DATA [file supp_gkv502_Supplemental_information10.pdf]

Supplemental information for:

Splicing of a non-coding antisense transcript controls *LEF1* gene expression.

Manuel Beltran, Estel Aparicio-Prat, Rocco Mazzolini, Alba Millanes-Romero, Pere Massó, Richard G. Jenner, Víctor M. Díaz, Sandra Peiró, and Antonio García de Herreros

Supplementary Methods

Reagents, cell culture and generation of transfectants. All cells were grown in Dulbecco's modified Eagle's medium (Invitrogen) containing 10% fetal calf serum (Biological Industries) and the standard complements. The generation of HT-29 M6 Snail1, RWP-1 Snail1 or SW-480 E-cadherin cells has been reported (1, 2). RWP-1 and HT-29 M6 cell populations were also transfected with pBabe-unspliced *LEF1* NAT plasmid or with an empty plasmid using a Lipofectamine Plus kit (Gibco) according to the manufacturer's instructions. Transfected cells were grown in medium containing 2.5 mg/ml of puromycin (Gibco).

The following antibodies were used in this study: anti-H3K4me2 (07-030 Millipore), anti-H3K27me3 (Ls-069-100 Diagnode), anti-Suz12 (2A09, Active Motif), anti-EzH2 (BD43, Millipore), anti-biotin (Sigma), anti-LEF1 (Cell Signalling, 22305), anti-HA (Sigma, H6908) and anti Pyruvate Kinase (PyrK) (Chemicon, AB1235).

Transcript analysis. RNA extraction was performed using Trizol (Invitrogen) and samples were treated with DNAse *Turbo* (Ambion) to avoid DNA contamination.Transcripts were analyzed by RT–PCR, using 0.5–1 µg of total RNA. The semi-quantitative analysis of the transcripts was performed using the One Step

RT–PCR kit (Qiagen) with the oligos indicated in Table S1. Expression of LEF1 antisense transcripts NAT was detected with the oligos showed in this table; in this case, the reaction was performed in two steps, using the reverse oligonucleotide in the retro-transcription reaction and the forward oligo as the primer for the PCR. All amplified fragments were verified by sequencing.

Where indicated, expression levels of transcripts were also determined by retrotranscription and real-time PCR (qRT-PCR), using the Transcriptor First Strand cDNA Synthesis kit (Roche) and the LightCycler ® 480 Real-Time PCR System (Roche). RNA levels were determined quantitatively in triplicate on a LightCycler 480 system. The relative quantification value for each target gene compared with the calibrator for that target is expressed as 2-(Ct-Cc) (where Ct and Cc are the mean threshold cycle differences after normalizing to HPRT expression).

The quantification of the number of NAT molecules per cell was determined as by qRT-PCR using a LEF1-NAT RNA (NAT +213/-1856) generated by *in vitro* transcription (see below) as Standard. The qRT-PCR analysis (fragment -11/-113) was performed with serial dilutions of RNA obtained from a known number of RWP-1 cells and compared with different amounts of the *in vitro* transcribed RNA.

For RNA isolation from nuclear and cytoplasmic compartments, RWP-1 cells were harvested, washed once with cold PBS and centrifuged at 4ºC for 5 minutes at 1200 rpm. The pellets were resuspended with a soft lysis buffer (50 mM Tris HCl pH 8; 10 mM EDTA; 0,1% NP-40; 10% Glycerol) and centrifuged at 4ºC for 15 minutes at 3000 rpm. The supernatant, containing the cytoplasmic fractions, were immediately transferred to a new tube and mixed with Trizol Reagent (Life Technology) to avoid RNA degradation. The pellet, containing the nuclear fraction, were washed once with the soft lysis buffer and then resuspended in SDS lysis buffer (1% SDS; 10 mM EDTA; 50 mM Tris pH 8) by pipetting several times. After incubating on ice for 15 minutes, Trizol reagent was added. RNA extraction of the cytoplasmic and the nuclear fractions were carried out following the manufacturer's protocol. The RNAs were retro-

transcribed into cDNA using Transcritpor first strand cDNA synthesis kit (Roche), and the relative amount was assessed by qPCR. *HPRT* and *U6* snRNA were used as cytoplasmic or nuclear-enriched controls.

***In vitro* transcription.** The different LEF1-NATs fragments were cloned into pcDNA3 under the control of the T7 polymerase promoter. The plasmids were then linearized and the different LEF1-NATs RNAs were transcribed *in vitro* using the Megascript T7 transcription kit (Life Technologies) following the manufacturer's instructions. The transcribed fragments of the NAT corresponded to +213/-1856, -754/-1856, -387/-1856, +1/-405, +1/-879, +1/-442, and +1/-1463. When indicated, the RNAs were biotinylated by addition of bio-14-dUTP (40% of the total dUTP) to the reaction. The control transcripts *YB1X* (NM004559), *Cre* (YP006472), or an RNA transcript generated from pcDNA3 were used. After synthesis, RNA was treated with DNase I (Ambion), purified with phenol-chloroform and precipitated with isopropanol. The integrity of the resultant RNAs was checked in 1% formamide-agarose gels.

Luciferase reporter assay. Reporter assays were performed using 100-400 ng of the indicated constructs containing the different promoters cloned into the pGL3 plasmid. Transfections were performed as previously reported (1) using Lipofectamine (Gibco). When appropriate, cells were also transfected with the NAT constructs inserted into the pcDNA3 plasmid. 10 ng of Renilla luciferase was also co-transfected as a control for transfection efficiency. Firefly and Renilla luciferase activities were analyzed 48 h after transfection, according to the manufacturer's instructions.

Alternatively the expression of luciferase mRNA and NAT was determined by qRT-PCR. RNA was extracted with Trizol Reagent (Life Technology) and treated with DNAseI (Ambion). The expression of Firefly and Renilla luciferases was determined by qRT-PCR using oligo-dT primer (Life Technologies) in the RT-PCR and the following Firefly-Luc: Sense, 5'-GCACATATCGAGGTGGACATC-3'; Antisense, 5'-

CGCAACTGCAACTCCGAT-3’); Renilla-Luc: Sense, 5’-GAATCGGATCCAGGATTCTT-3’; Antisense: 5’-TCTTGCGAAAAATGAAGACC-3’. Transcription of the antisense strand in the pGL3 constructs was quantified using the strand-specific primer NAT -113 (5’-GCACGAACCCTTCCAACTCT-3’) in the RT reaction, and the primers NAT -113 and NAT -11 (5-’TCTGTAATCTCCGCTCCGCT-3’) in the qPCR.

Nuclear Run-On Assay. Thirty million cells were collected, centrifuged at 270 xg at 4ºC and washed twice with PBS. Nuclei were purified by ultracentrifugation through a sucrose cushion. Briefly, the cell pellet was resuspended in 4 ml ice-cold Sucrose Buffer I (0.32 M sucrose, 3 mM $CaCl_2$, 2 mM magnesium acetate, 0.1 mM EDTA, 10 mM Tris HCl, pH 8.0, 1 mM DTT, 0.5% (v/v) Nonidet P-40). A small aliquot of cells was examined for completion of the lysis with a phase-contrast microscope. Then, 4 ml Sucrose Buffer II (2 M sucrose, 5 mM magnesium acetate, 0.1 mM EDTA, 10 mM Tris HCl, pH 8.0, 1 mM DTT) were added. The nuclei suspension was mixed by gentle inversion and carefully layered onto a sucrose cushion (consisting in 4.4 ml Sucrose Buffer II in a polyallomer SW 40.1 tube). The gradient was centrifuged 45 minutes at 30,000 xg at 4ºC; the supernatant was removed by vacuum aspiration and the nuclei pellet was resuspended in 100 µl ice-cold glycerol storage buffer (50 mM Tris HCl, pH 8.3, 40% glycerol, 5 mM $MgCl_2$, 0.1 mM EDTA). In vitro RNA synthesis was performed by gently adding one volume of transcription buffer (200 mM KCl, 20 mM Tris HCl, pH 8.0, 5 mM $MgCl_2$, 4 mM DTT, 4 mM each of ATP, GTP and CTP, 200 mM sucrose and 20% glycerol), supplied with 0.8 mM biotin-16-UTP (Enzo Lifescience). The mixture was incubated for 30 minutes at 30ºC. Then, 60 units of RNase-free DNase I were added and incubated for 15 minutes at 30ºC. RNA was purified with TRIZOL reagent (Life Technologies) according to the manufacturer’s instructions and resuspended in 50 µl DEPC-treated water. The biotinylated RNA was bound to 50 µl magnetic streptavidin beads (New England BioLabs) resuspended in binding buffer (10 mM Tris-HCl, pH 7.5,

1 mM EDTA and 2 M NaCl) by incubating during 20 minutes at 42ºC and 2 hours at room temperature. To monitor undesired RNA capture to the streptavidin beads, a control reactions was set up in which UTP was added to the mixture in the place of biotin-16-UTP. Beads were rescued with a magnetic apparatus and washed twice for 15 minutes with washing buffer (15% formamide and 2x standard saline citrate SSC), followed by a 5-minutes washing in 1 mL 2x SSC. Beads were then resuspended in 30 μl DEPC-treated water. Random hexamer primed cDNA was prepared from 10 μl run-on RNA-beads (Transcriptor First Strand CDNA Synthesis Kit – Roche). Then, the cDNA was separated by the magnetic beads and the levels of transcription of LEF1, LEF1-NAT and the control RNA were quantified by qPCR as previously described.

Chromatin Immunoprecipitation (ChIP) assay. ChIP assays were performed as previously described (3). Cells (1 or 2 $\times 10^7$) were crosslinked with 1% formaldehyde for 10 min. Cells were lysed in IP1 buffer (50 mM Tris HCl, pH 8, 10 mM EDTA, 0.1% NP-40, and 10% glycerol) for 10 min at 4ºC. The pellet obtained was then lysed in IP2 buffer (50 mM Tris HCl, pH 8, 10 mM EDTA, and 1% SDS). Samples were sonicated ten times at 40% for 10 seconds (Branson), to generate 200- to 500-bp DNA fragments. After immunoprecipitation with the indicated antibodies in IP buffer (16.7 mM Tris HCl, pH 8, 167 mM NaCl, 1.2 mM EDTA, 1.1% Triton X-100, 0.01% SDS), samples were treated with elution buffer (100 mM Na_2CO_3, 1% SDS), and incubated at 65°C overnight in elution buffer plus 200 mM NaCl to reverse the formaldehyde crosslinking. After treating for 1h with proteinase K (Roche), DNA was purified using a GFX PCR DNA and gel band purification kit (Amersham).

Promoter regions were detected by quantitative PCR with the LightCycler ® 480 Real-Time PCR System (Roche), using the following oligos: 5’-AACTCTCTTTTCCTTGTCCTTCTG-3’ and 5’-GCAGAGGGAGGAAGATGAAA-3’ for the -1806/-1628 amplicon; 5’-AGACTCGTCCTACAGGATCTGG-3’ and 5’-CGCTGAAAAGCTACCCACTT-3’ for the -1360/-1188 amplicon; 5’-

ACTGAGTGTGTGTGTCGGCT-3’ and 5’-ATCTGCTAGAGAAGGAGGAGGAG-3’ for the -904/-703 amplicon; 5’-CTCGAGCCGGGAACAAAGA-3’ and 5’-GGGAAGAGAAAGAGAAGTTTGCC-3’ for the -951/-750 amplicon (all corresponding to the *LEF1* promoter); 5’-CAGAGAGGGAGGAAGGGAAC-3’ and 5’-CCCCTCTACCTCCCATCCTA-3’ for +266/+433 amplicon (corresponding to the NAT promoter); 5’-CATCTGGTTTGCTGCTAAGCTA-3’ and 5’-CAATGATGCACTGACTTCCCTTT-3’ for the +3864/+4048 amplicon used as a control sequence; and 5’-ACCAGGGATTTCAGTCGATGTA-3’ and 5’-CAGGCAGTTCTATGAGGCAGAG -3’ for the +483/+657 amplicon in the Luciferase, also used as control. PCR and data collection were performed with the LightCycler 480 system.

Analysis of cell migration and proliferation. Proliferation studies were performed seeding equal amount of cells at low confluence in a 96 well plate. Once cells attached, the plate was incubated in Incucyte Essen (Essen Bioscience) where three high-definition phase contrast images of the same area were taken each two hours. Using Incucyte Essen software, the images were analyzed in order to calculate the average confluence in each image, and represented as the average of three different replicates at the different time points. Error bars indicate standard error of mean (SEOM).

Two-dimension cell invasion assays were performed seeding the cells at confluence in 24-well plate. Once cells attached, a scratch was performed using Essen 24-well wound maker (Essen Bioscience). Cells were washed three times with PBS and the plate was incubated with complete media in Incucyte Essen (Essen Bioscience) as indicated above. Images were analyzed measuring the changes of position of the cell frontline in each image and represented as average of three different replicates at the different time points.

Analysis of NAT-bound proteins by mass spectrometry. A biotinylated RNA corresponding to the LEF1-NAT +213/-1856 was transcribed *in vitro*. As a negative control, the transcript from the empty pcDNA3 vector was used. RWP1 cells were lysed with soft lysis buffer (50 mM Tris HCl pH 8, 10 mM EDTA, 0,1% NP-40, 10% glycerol) supplemented with protease inhibitors. Lysate was brought to 150 mM salt concentration. 750 µg of the cell lysate were precleared by incubating with 20 µl of magnetic streptavidin beads at 4ºC for 2 hours on a rotating wheel. Meanwhile, 5 µg of biotynilated RNA were heated to 90°C for 2 minutes, cooled on ice for 2 minutes, brought up to 50 µl with RNA structure buffer (10 mM Tris HCl, pH 8, 100 mM KCl, 10 mM $MgCl_2$) and then shifted to room temperature for 20 minutes to allow proper secondary structure formation. The biotynilated RNA was added to the pre-cleared cell lysate and incubated at 4ºC overnight on a rotating wheel. The next day, 5 µg of magnetic streptavidin beads were added and incubated at 4ºC for 4 hours. The beads were then rescued with a magnetic rack and washed with the following buffers (4 times each): low salt washing buffer (20 mM Tris HCl pH 8, 5 mM NaCl, 2 mM EDTA 0.1% SDS, 1% Triton X-100), high salt washing buffer (20 mM Tris HCl pH 8, 150 mM NaCl, 2 mM EDTA 0.1% SDS, 1% Triton X-100) and LiCl buffer (10 mM Tris HCl pH 8, 1 mM EDTA, 250 mM LiCl, 15 NP-40, 1% sodium deoxycholate). The beads were then eluted with 30 µl of urea 6M/ABC 100mM at 37ºC for 30 minutes and the eluted material was stored at -20ºC until mass spectrometry analysis was performed.

A duplicate set of this samples was eluted in SDS-PAGE sample buffer (62.5 mM Tris HCl pH 6.8; 2.5 % SDS; 0.002 % Bromophenol Blue; 0.7 M (5%) β-mercaptoethanol; 10 % glycerol) at 95ºC for 5 minutes and loaded on a 10% polyacrylamide gel. Suz-12 expression was quantified by western blotting.

For mass spectrometry , samples were prepared as follows. Samples were reduced with ditiothreitol (227 nmols, 1 h, 37°C) and alkylated in the dark with iodoacetamide (454 nmols, 30 min, 25 ºC). The resulting protein extract was then diluted with 200 mM NH_4HCO_3 (1/6) and digested with 7 µg LysC (Wako, cat # 129-

02541) overnight at 37ºC and then with 7 μg of trypsin (Promega, cat # V5113) for eight hours at 37˚C.

Seven μg of each sample was analyzed using a LTQ-Orbitrap XL mass spectrometer (Thermo Fisher Scientific, San Jose, CA, USA) coupled to an EasyLC (Thermo Fisher Scientific (Proxeon), Odense, Denmark). Peptides were loaded directly onto the analytical column at a flow rate of 1.5-2 µl/min using a wash-volume of 4 times the injection volume, and were separated by reversed-phase chromatography using a 25-cm column with an inner diameter of 75 µm, packed with 5 µm C18 particles (Nikkyo Technos Co., Ltd. Japan). Chromatographic gradients started at 93% buffer A and 7% buffer B for 7 minutes with a flow rate of 250 nl/min, and gradually increased to 65% buffer A and 35% buffer B in 60 min. After each analysis, the column was washed for 15 min with 10% buffer A and 90% buffer B. Buffer A: 0.1% formic acid in water. Buffer B: 0.1% formic acid in acetonitrile.

The mass spectrometer was operated in positive ionization mode with nanospray voltage set at 2.5 kV and source temperature at 200 °C. Ultramark 1621 for the FT mass analyzer was used for external calibration prior the analyses. Moreover, an internal calibration was also performed using the background polysiloxane ion signal at m/z 445.1200. The instrument was operated in DDA mode and full MS scans with 1 micro scans at resolution of 60,000 were used over a mass range of m/z 350-1500 with detection in the Orbitrap. Auto gain control (AGC) was set to 1E6, and dynamic exclusion (60 seconds) and charge state filtering disqualifying singly charged peptides were both activated. In each cycle of DDA analysis, following each survey scan the top twelve most intense ions with multiple charged ions above a threshold ion count of 5000 were selected for fragmentation at normalized collision energy of 35%. Fragment ion spectra produced via collision-induced dissociation (CID) were acquired in the ion trap, AGC was set to 5E4, isolation window of 2.0 m/z and maximum injection time of 50 ms was used. All data were acquired with Xcalibur software v2.2.

Data Analysis. Proteome Discoverer software suite (v1.4, Thermo Fisher Scientific) and the Mascot search engine (v2.5, Matrix Science (4)) were used for peptide identification and quantification. The data were searched against a SwissProt database containing entries corresponding to Homo sapiens (version of January 2014), a list of common contaminants, and all the corresponding decoy entries. Trypsin was chosen as enzyme and a maximum of three miscleavages were allowed. Carbamidomethylation (C) was set as a fixed modification, whereas oxidation (M) and acetylation (N-terminal) were used as variable modifications. Searches were performed using a peptide tolerance of 7 ppm, a product ion tolerance of 0.5 Da. Resulting data files were filtered for FDR < 5 %.

References

1. Batlle E, Sancho E, Francí C, Domínguez D, Monfar M, Baulida J, García de Herreros A (2000). The transcription factor snail is a repressor of E-cadherin gene expression in epithelial tumour cells. *Nat Cell Biol,* **2**, 84-89.

2. Solanas G, Porta-de-la-Riva M, Agustí C, Casagolda D, Sánchez-Aguilera F, Larriba MJ, Pons F, Peiró S, Escrivà M, Muñoz A, et al. (2008). E-cadherin controls beta-catenin and NF-kappaB transcriptional activity in mesenchymal gene expression. *J Cell Sci*, **121**, 2224-2234.

3. Peiró S, Escrivà M, Puig I, Barberà MJ, Dave N, Herranz N, Larriba MJ, Takkunen M, Francí C, Muñoz A, et al. (2006). Snail1 transcriptional repressor binds to its own promoter and controls its expression. *Nucleic Acids Res,* **34**, 2077-2084.

4. Perkins D N, Pappin DJ, Creasy D M, Cottrell JS (1999) Probability-based protein identification by searching sequence databases using mass spectrometry data. *Electrophoresis* **20**, 3551-3567.

Supplementary Table 1

Gene	Position in *LEF1* locus	Oligonucleotide	
HPRT		Forward	5’-GGCCAGACTTTGTTGGATTTG-3’
		Reverse	5’-TGCGCTCATCTTAGGCTTTGT-3
Pumilio		Forward	5’-CGGTCGTCCTGAGGATAAAA-3’
		Reverse	5’-CGTACGTGAGGCGTGAGTAA-3’
LEF1 mRNA	+132/+2614	Forward	5’-CGAAGAGGAAGGCGATTTAG-3’
		Reverse	5’-GTCTGGCCACCTCGTGTC-3’
	+3864/+4048	Forward	5’-CATCTGGTTTGCTGCTAAGCTA-3’
		Reverse	5’-CAATGATGCACTGACTTCCCTTT-3’
LEF1 NAT	-11/-8596	Forward	5’-TCTGTAATCTCCGCTCCGCT-3’
		Reverse	5’-CACTGTGCCTGTGTAGGATGTG-3’
	-11/-391	Forward	5’-TCTGTAATCTCCGCTCCGCT-3’
		Reverse	5’-GCGTGCTCCCTCCAGAA-3’
	-11/-113	Forward	5’-TCTGTAATCTCCGCTCCGCT-3’
		Reverse	5’-GCACGAACCCTTCCAACTCT-3’
	-11/-5751	Forward	5’-TCTGTAATCTCCGCTCCGCT-3’
		Reverse	5' - GGAAGAGAACCCTTGGCCTT-3'
	-769/-1856	Forward	5’-TTGCCAAGAATAAAGTTTTTGCC-3’
		Reverse	5’-GTACCCTTGTCTCCAAAGAGCG-3’
	+4/-953	Forward	5’-TCCCGGCGGCTCTG-3’
		Reverse	5’-GCCGGCGAGCCAGG-3’
	+213/+60	Forward	5’-CCTCGTGTCCGTTGCTG-3’
		Reverse	5’-GACGAGATGATCCCCTTCAAG-3’
	-1562/-3057	Forward	5’-CACTCCTTTTCCTCTGCCAGTC-3’
		Reverse	5’-CTGCATTTTCCACTTGTTTTCC-3’
	-2950/-4550	Forward	5’-CAGAGAGGTGAACGTGTAGCTG-3
		Reverse	5’-GCCTCTGGAGTCACATTATTCG-3’
	-4004/-4850	Forward	5’-CAATTGCTAGGGGCTGGCT-3’
		Reverse	5’-CCGAGCTACAGACGCCAA-3’
	+4/-1856	Forward	5’-TCCCGGCGGCTCTG-3’
		Reverse	5’-GTACCCTTGTCTCCAAAGAGCG-3’
	-1562/-1688	Forward	5’-CACTCCTTTTCCTCTGCCAGTC-3’
		Reverse	5’-CTCCCCACTGTCAGAGCATCT-3’
	-5653/-8596	Forward	5' - CCCCTTTGTGTGACTAAATTTGG-3'
		Reverse	5’-CACTGTGCCTGTGTAGGATGTG-3’
	-7831/-7933	Forward	5’-GGTCTCAAATTTCAAACCTCAGG-3’
		Reverse	5’-GGACATTTGGGAAAAGGTACATTG-3’
	-7831/-8228	Forward	5’-GGTCTCAAATTTCAAACCTCAGG-3’
		Reverse	5’-CCATATCCACCCAGGAAAGAG-3’
	-8098/-8822	Forward	5’-GGCAATGGAGAAACCCTGA-3’
		Reverse	5’-AACAGAAAGCATCCTCAAGGTCA-3’
	-8661/-9495	Forward	5’-AGGATTGAGCCTCCTTTTTCTCA-3’
		Reverse	5’-TTCACACCACTGCACTCCAG-3’
	-9385/-10157	Forward	5’-CTCTCTCACTGTTAGCCCTCCC-3’
		Reverse	5’-AATAGGCCAAGCAGTGACAG-3’
	-10032/-10647	Forward	5’-GGTTCTGCTCAGATAGTCCCTCCT-3’
		Reverse	5’-CTTGCTGTAAGTAAACTGCACTG-3’
	-10438/-11115	Forward	5’-CTGTAATTATGCAGATCAGCACTG-3’
		Reverse	5’-ATGTGTAAGGCAATATGGGGG-3’
	-11100/-11482	Forward	5’-CAGCAACTTACAGCACGATTCAG-3’
		Reverse	5’-GGTTTGGCAGTTTTACAGGGAG-3’
	-11334/-11785	Forward	5’-GTTGTGGTTCTCGTGTGTTGC-3’
		Reverse	5’-TCCAGAAAATGACCCTGCTCT-3’
	-11716/-12252	Forward	5’-TGCAAAAGTCATTCCCCTCCA-3’
		Reverse	5’-ACTTCACTGAAAATCTATGGTTCAC-3’

	-13237/-13475	Forward	5'-TGGAAAGGTCTAGCTGTGGA-3'
		Reverse	5'-AAACAAAACAGTGCCCCTGC-3'
	-13719/-14138	Forward	5'-TCCCAGGTTCAAGCGATTCT-3'
		Reverse	5'-GCCCTGAAATTGTGTCTGCT-3'
	-14119/-14531	Forward	5'-AGCAGACACAATTTCAGGGC-3'
		Reverse	5'-TCTCAGAGCTGCCCGATTTA-3'
	-14718/-15109	Forward	5'-TTTTCTTGCTGGTTGCTTCGG-3'
		Reverse	5'-TTCCCAAGTCTCTGGCACAC-3'
	-15109/-15504	Forward	5'-ACATGAGGCTCTTTGGTGGG-3'
		Reverse	5'-GCATGGACCTTTGAGCCCAT-3'
	-15488/-15955	Forward	5'-GGCTCAAAGGTCCATGCCAA-3'
		Reverse	5'-AACTTACGTAGCCACCAGGC-3'
	-15936/-16426	Forward	5'-GCCTGGTGGCTACGTAAGTT-3'
		Reverse	5'-AGTGCTGGGAGCCCTAAGAT-3'
	-16416/-16877	Forward	5'-CTCCCAGCACTGTGGAAGAA-3'
		Reverse	5'-GTGTTCACAGTGAGGCTTGC-3'
	-17021/-17430	Forward	5'-ATTGGTGCCTCACCAGAAGAG-3'
		Reverse	5'-TGGAGAGCTGGGGCAAAAAG-3'
	-17395/-17816	Forward	5'-CCACATTCCAGGCATGCTTT-3'
		Reverse	5'-AGAGGTTTGTAGGCAGCTGT-3'
	-17797/-18188	Forward	5'-ACAGCTGCCTACAAACCTCT-3'
		Reverse	5'-AATTAGCTGGGTGTGGTGGT-3'
Zeb2		Forward	5'-ATAAGGGAGGGTGGAGTGGA-3'
		Reverse	5'-CGCGTTCCTCCAGTTTTCTT-3'
Claudin4		Forward	5'-TGTCCCCGAGAGAGAGTGCCCT-3'
		Reverse	5'-GGGGCCGTAGGATTCCAAGCG-3'
CDH1 (E-cadherin)		Forward	5'-GAACGCATTGCCACATACAC-3'
		Reverse	5'-ATTCGGGCTTGTTGTCATTC-3'
Occludin		Forward	5'-ACAATCAGCCATGTCATCCAGG-3'
		Reverse	5'-CTGGAGGAGAGGTCCATTTGTAG-3'
Zeb1		Forward	5'TTCACAGTGGAGAGAAGCCA-3'
		Reverse	5'-GCCTGGTGATGCTGAAAGAG-3'
U6 snRNA		Forward	5'-CGGCAGCACATATACTAAAATTGG-3'
		Reverse	5'-AAAAATATGGAACGCTTCACGAA-3'

Supplementary Table 1. The pairs of oligonucleotides used for RT-PCR analysis of the different transcripts are shown.

Supplementary Figure 1

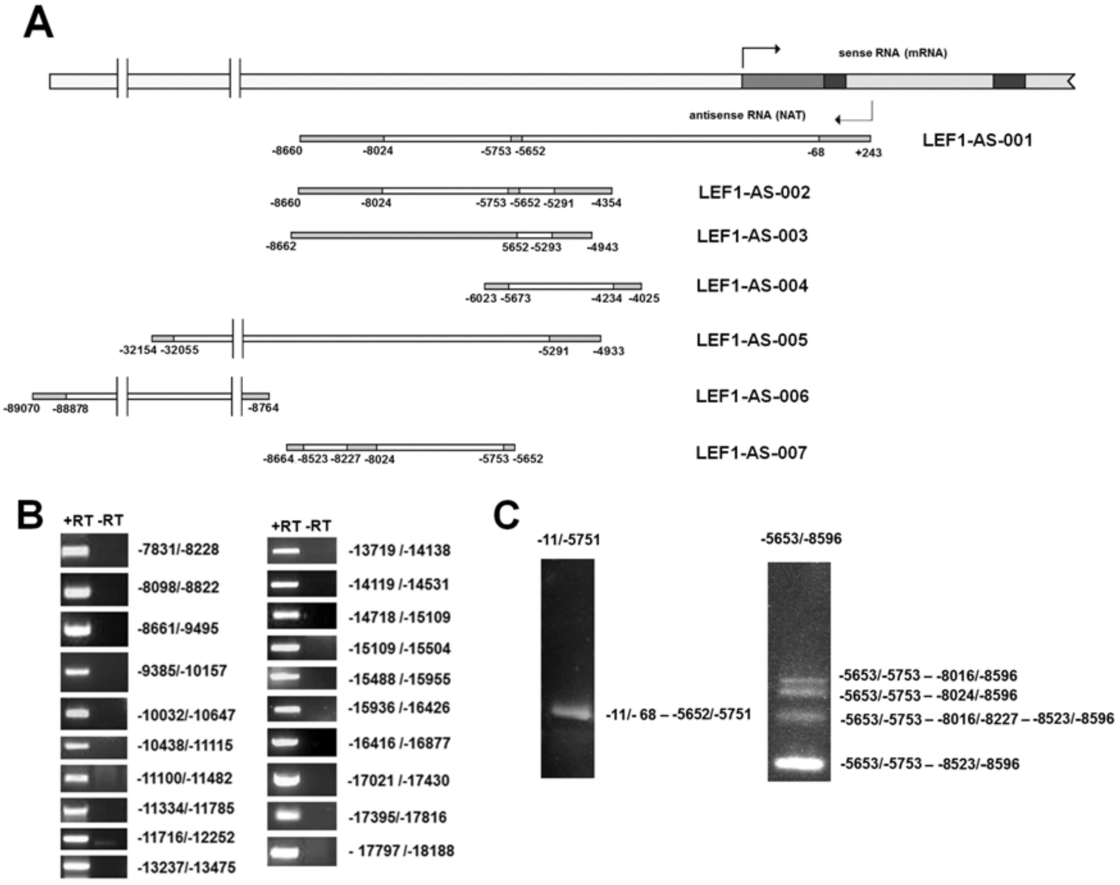

Supplementary Figure 1. ***LEF1* antisense transcripts detected in the *LEF1* locus**. The Ensemble database refers to seven *LEF1* antisense transcripts already sequenced. They are depicted in panel **A** and referenced to the main transcription start site of the *LEF1* mRNA. Only *LEF1-AS-001* overlaps with a significant part of the *LEF1* promoter and will be studied in this work. The described distribution of exons (in grey) and introns is presented; note that the spliced *LEF1-AS-001* detected in our assays corresponded to a variant of above shown form with a shorter third exon comprising only -8523/-8660. As presented in panel **B**, antisense transcription in this locus was detected until -18100 using strand-specific RT-PCR. Other not-reported spliced variants of these transcripts containing exons -8016/-8227 and -8016/-8660 were also observed (panel **C**).

Supplementary Figure 2

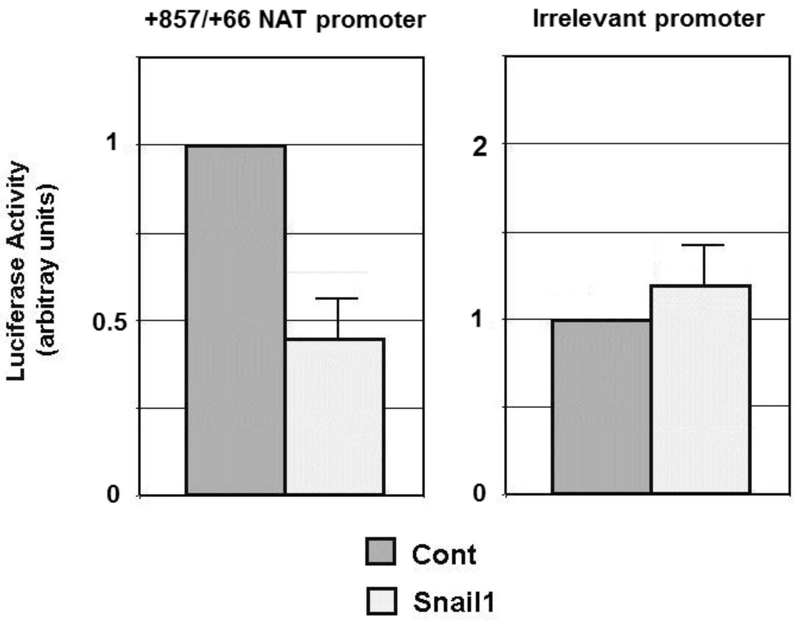

Supplementary Figure 2. **A *LEF1* NAT promoter directs the expression of *LEF1* NAT.** A +66/+857 fragment of the *LEF1* gene was inserted in the opposite orientation into the pGL3 plasmid. The promoter activity of this DNA fragment was analyzed in RWP-1 cells stably transfected with either the control plasmid or with pcDNA3-Snail1. A 300 bp fragment of the *HES* promoter (a kind gift of Dr A. Bigas, IMIM, Barcelona, Spain) was used as control. Values refer to those obtained in control cells. The average ± SD of three experiments performed in triplicate are shown.

Supplementary Figure 3

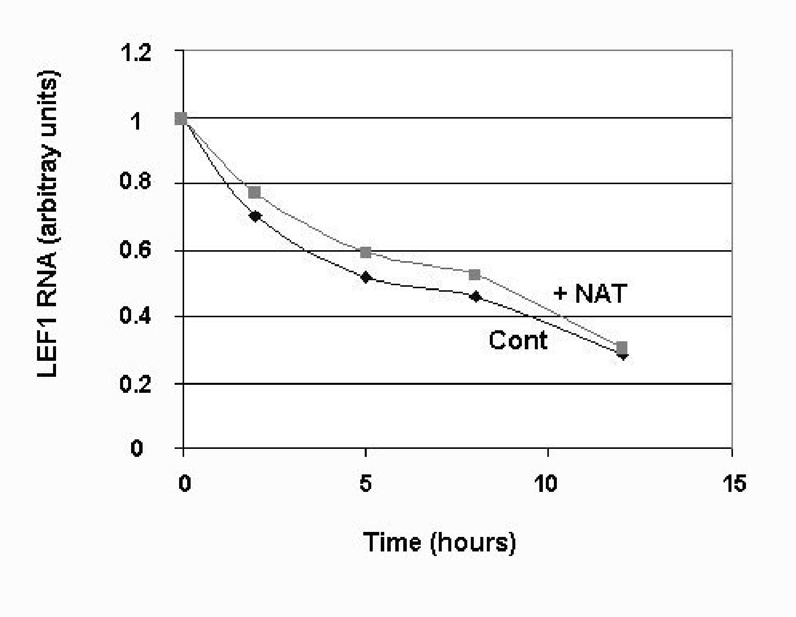

Supplementary Figure 3. ***LEF1* NAT does not affect *LEF1* mRNA stability**. RWP-1 Snail1 cells, transfected with pBabe LEF1 NAT (+58/-1856) or empty pBabe, a were supplemented with actinomycin D (2 μg/mL) to inhibit transcription. RNA was collected at the indicated times after actinomycin D addition and *LEF1* mRNA levels were determined by qRT-PCR. The relative amount of this RNA with respect to the initial time is shown. The figure shows the results of one experiment of two performed with almost identical results.

Supplementary Figure 4

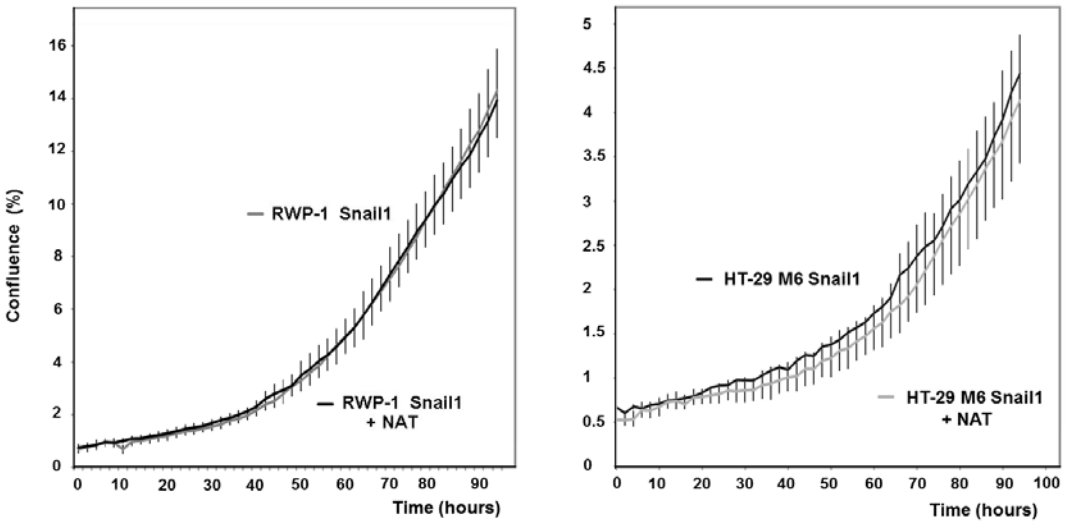

Supplementary Figure 4. **Unspliced LEF1 NAT does not affect cell proliferation.** Proliferation assays were performed as indicated in Suppl Methods with HT-29 M6 or RWP-1 cell stably transfected with pcDNA3 encoding unspliced NAT or empty plasmid. Average of three independent replicates ± SD is presented in the figure.

Supplementary Figure 5

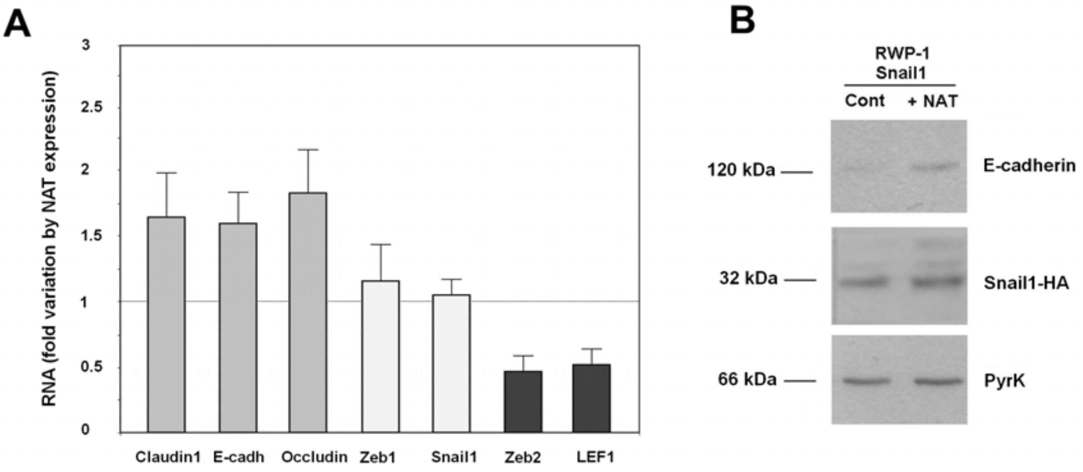

Supplementary Figure 5. **NAT expression in RWP-1 Snail1 cells only slightly increases E-cadherin expression.** The mRNA (**A**) or protein (**B**) levels of the indicated genes were determined as indicated in Methods in unspliced NAT-transfected RWP-1 Snail1 cells and compared with the same cells transfected with the control plasmid. The average ± SD of three independent experiments (A) or the result of a representative experiment is presented.

Supplementary Figure 6

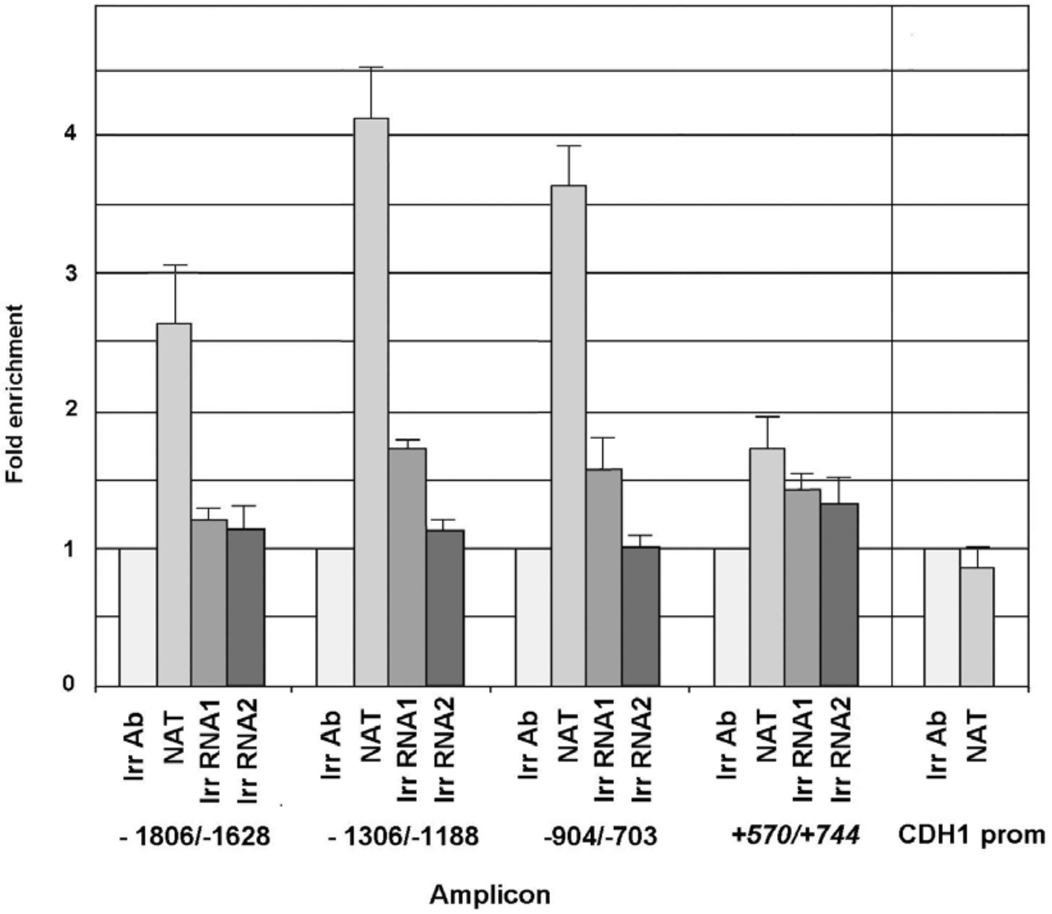

Supplementary Figure 6. ***LEF1* NAT binds to *LEF1* promoter**. RWP-1 cells were transfected with pGL3-LEF1 promoter and *in vitro* synthesized biotinylated unspliced NAT (+58/-1856) or irrelevant RNAs, corresponding to *Cre1* (IrrRNA1) or to a fragment of pcDNA3 plasmid (IrrRNA2). *CDH1* promoter was alternatively transfected when indicated. After 24h cells were fixed with formaldehyde as described for the ChIP assays, chromatins were prepared, incubated with an anti-biotin antibody and immunoprecipitated with protein-A-agarose. Presence of the indicated amplicons was carried out by qPCR as described in Methods. The results show the average ± SD of three experiments performed in triplicate.

Supplementary Figure 7

A

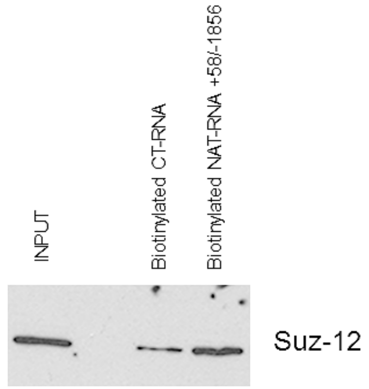

B

PROTEINS EXCLUSIVE OF NAT RNA

Description	Area CT RNA	Area NAT2kb
Ribonucleases P/ MRP protein subunit POP1 OS=Homo sapiens GN=POP1 PE=1 SV=2 – [POP1_HUMAN]	0,000E0	5,532E5
Pre-mRNA 3'-end-processing factor FIP1 OS=Homo sapiens GN=FIP1L1 PE=1 SV=1 - [FIP1_HUMAN]	0,000E0	4,347E5
Transcription intermediary factor 1-beta OS=Homo sapiens GN=TRIM28 PE=1 SV=5 - [TIF1B_HUMAN]	0,000E0	3,487E5
Mediator of RNA polymerase II transcription subunit 1 OS=Homo sapiens GN=MED1 PE=1 SV=4 - [MED1_HUMAN]	0,000E0	2,384E5
Pre-mRNA-splicing factor SPF27 OS=Homo sapiens GN=BCAS2 PE=1 SV=1 - [SPF27_HUMAN]	0,000E0	7,157E5
Mitochondrial tRNA-specific 2-thiouridylase 1 OS=Homo sapiens GN=TRMU PE=1 SV=2 - [MTU1_HUMAN]	0,000E0	8,004E5
Protein RRP5 homolog OS=Homo sapiens GN=PDCD11 PE=1 SV=3 - [RRP5_HUMAN]	0,000E0	5,425E5
Polypyrimidine tract-binding protein 1 OS=Homo sapiens GN=PTBP1 PE=1 SV=1 - [PTBP1_HUMAN]	0,000E0	4,884E5
60S ribosomal protein L32 OS=Homo sapiens GN=RPL32 PE=1 SV=2 - [RL32_HUMAN]	0,000E0	3,974E5
Zinc finger Ran-binding domain-containing protein 2 OS=Homo sapiens GN=ZRANB2 PE=1 SV=2 - [ZRAB2_HUMAN]	0,000E0	3,571E5
Probable ATP-dependent RNA helicase DDX47 OS=Homo sapiens GN=DDX47 PE=1 SV=1 - [DDX47_HUMAN]	0,000E0	3,216E5
Zinc finger CCCH domain-containing protein 4 OS=Homo sapiens GN=ZC3H4 PE=1 SV=3 - [ZC3H4_HUMAN]	0,000E0	1,767E5
Probable ATP-dependent RNA helicase DHX37 OS=Homo sapiens GN=DHX37 PE=1 SV=1 - [DHX37_HUMAN]	0,000E0	1,386E5
PHD and RING finger domain-containing protein 1 OS=Homo sapiens GN=PHRF1 PE=1 SV=3 - [PHRF1_HUMAN]	0,000E0	1,287E5
40S ribosomal protein S4, X isoform OS=Homo sapiens GN=RPS4X PE=1 SV=2 - [RS4X_HUMAN]	0,000E0	1,016E5
Transcriptional repressor CTCFL OS=Homo sapiens GN=CTCFL PE=1 SV=2 - [CTCFL_HUMAN]	0,000E0	9,797E4
Probable ATP-dependent RNA helicase DDX52 OS=Homo sapiens GN=DDX52 PE=1 SV=3 - [DDX52 HUMAN]	0,000E0	8,621E4
Nucleolar GTP-binding protein 2-OS=Homo sapiens GN=GNL2 PE=1 SV=1 - [NOG2_HUMAN] X52 HUMAN]	0,000E0	5,182E4

Supplementary Figure 7. **Bound proteins by unspliced NAT**. A biotinylated RNA corresponding to the LEF1-NAT +213/-1856 was transcribed *in vitro*. As a negative control, a 1.2 kb transcript from the empty pcDNA3 vector was used. The two RNAs

were incubated with a protein lysate obtained from RWP-1 cells as indicated in Methods; RNAs and associated proteins were purified using streptavidin beads; bound proteins were eluted and analyzed by mass spec. In parallel, the presence of PRC2 component Suz12 was determined in both fractions (**A**). Panel B shows a list with the proteins specifically detected in the NAT-bound fraction and not in the control, ordered by score. Notice that PRC2 components were not included in this list since, as shown in panel A, this complex also interacts with the irrelevant RNA although to a lower extent than to the NAT, and the list only contains proteins specifically bound to NAT.
