# Supplementary material for: Correction to ‘Splicing of a non-coding antisense transcript controls LEF1 gene expression’
Source: Nucleic Acids Res. 2026 Jul 14;54(13):gkag725. doi: 10.1093/nar/gkag725 (PMC13367179; doi:10.1093/nar/gkag725)
Supplement: gkag725_Supplemental_File [file gkag725_supplemental_file.pptx]

## Slide 1
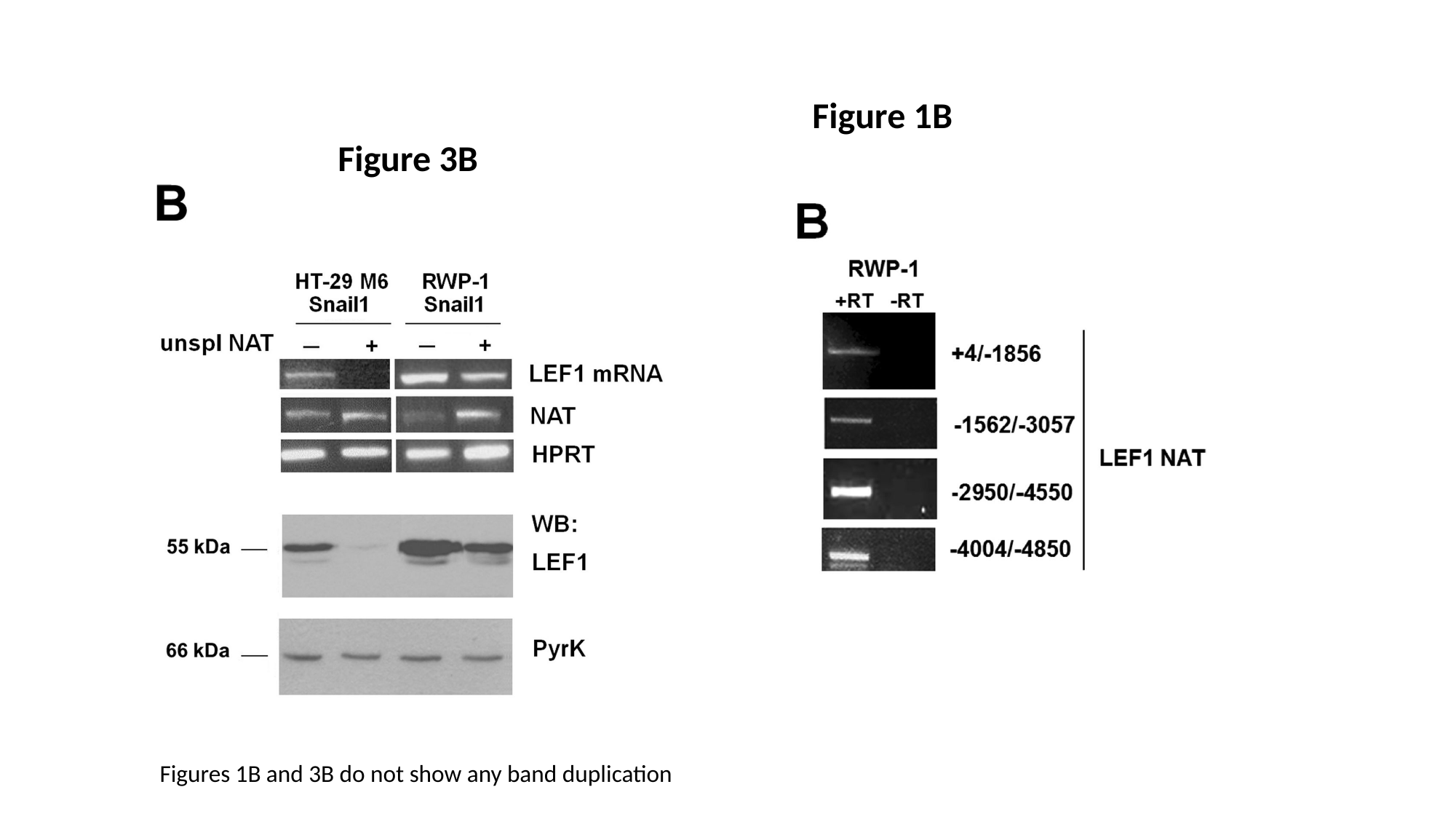

Figure 1B
Figure 3B
Figures 1B and 3B do not show any band duplication

## Slide 2
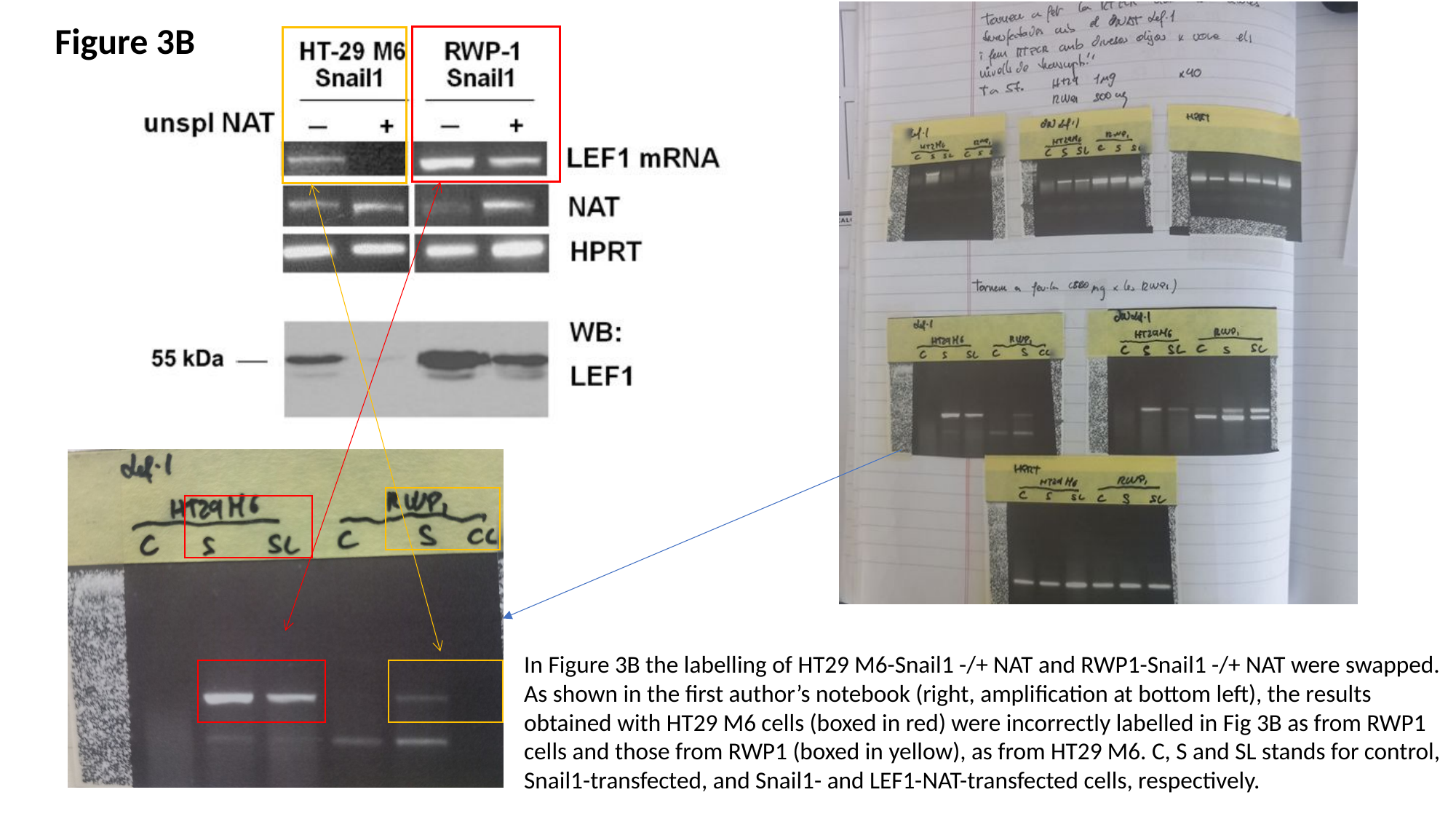

Figure 3B
In Figure 3B the labelling of HT29 M6-Snail1 -/+ NAT and RWP1-Snail1 -/+ NAT were swapped. As shown in the first author’s notebook (right, amplification at bottom left), the results obtained with HT29 M6 cells (boxed in red) were incorrectly labelled in Fig 3B as from RWP1 cells and those from RWP1 (boxed in yellow), as from HT29 M6. C, S and SL stands for control, Snail1-transfected, and Snail1- and LEF1-NAT-transfected cells, respectively.

## Slide 3
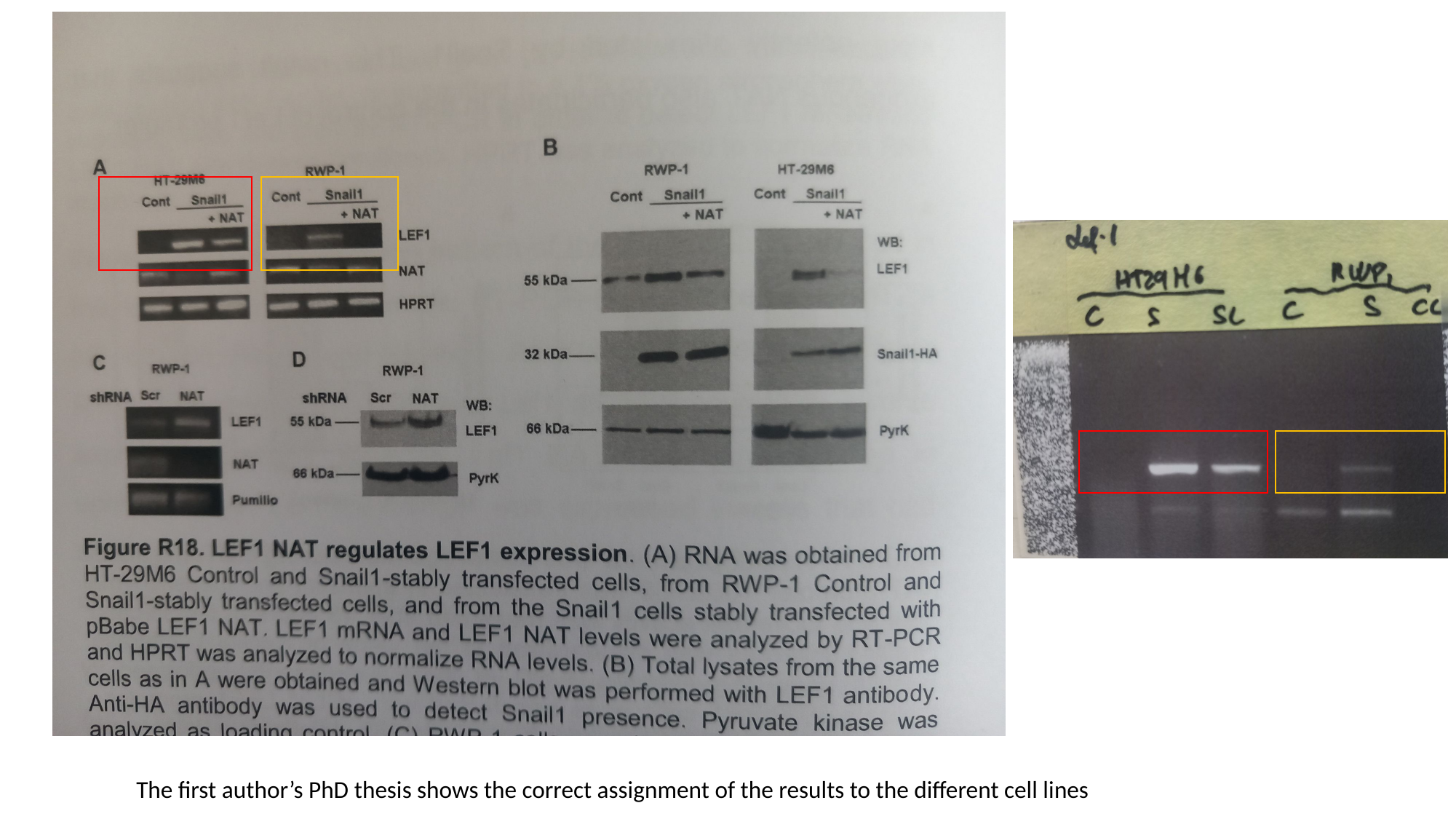

The first author’s PhD thesis shows the correct assignment of the results to the different cell lines

## Slide 4
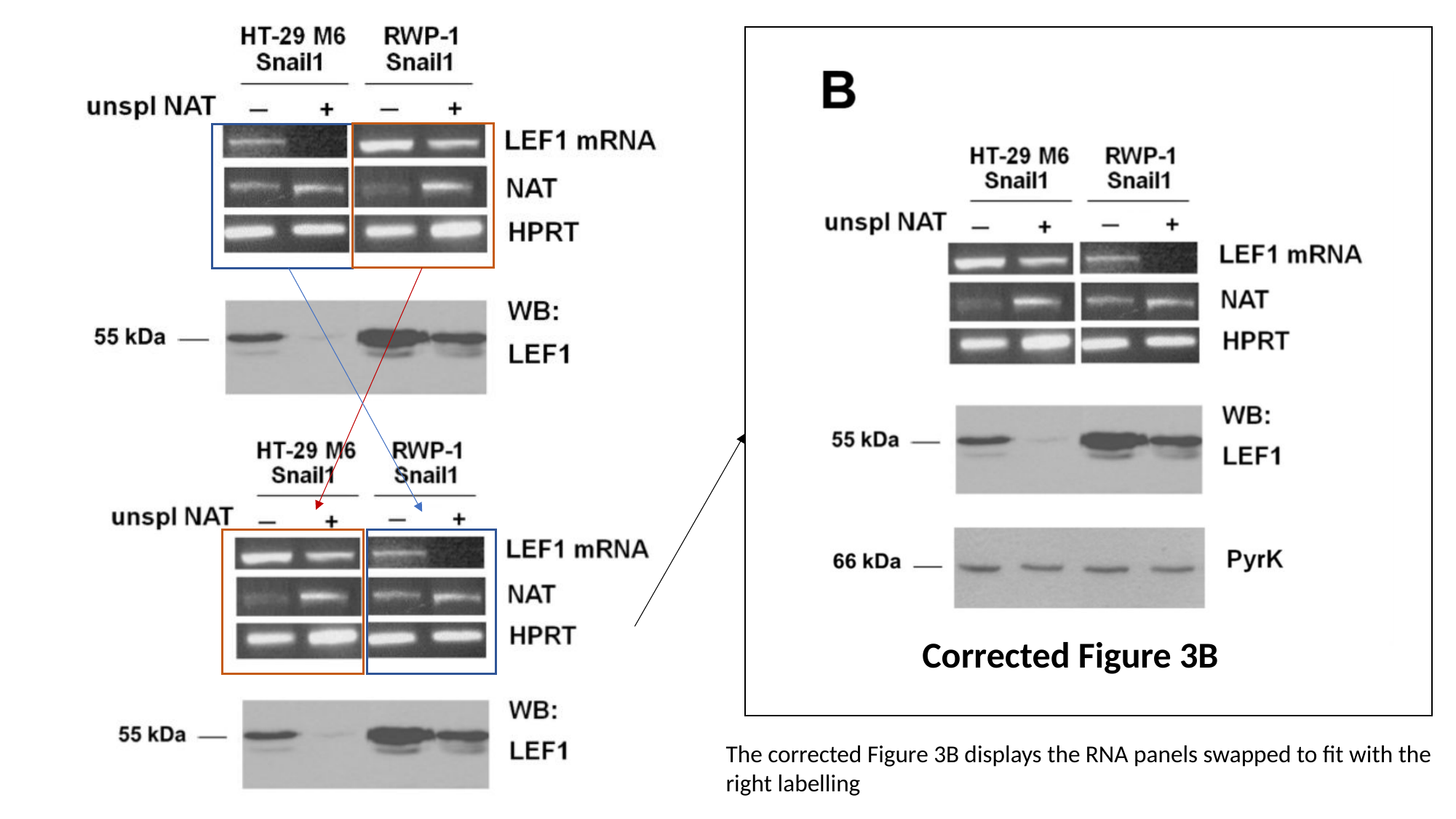

Corrected Figure 3B
The corrected Figure 3B displays the RNA panels swapped to fit with the right labelling

## Slide 5
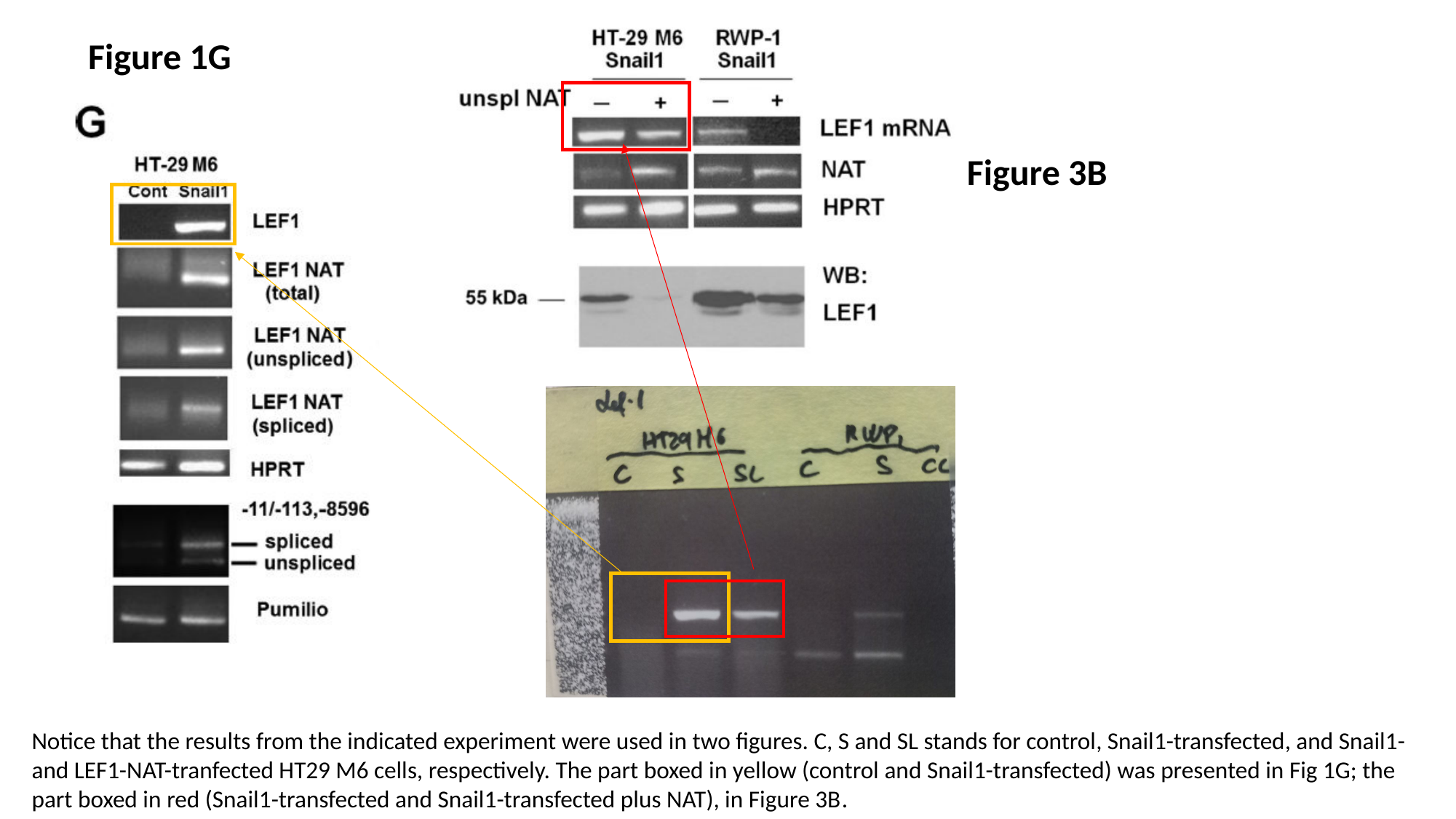

Figure 1G
Figure 3B
Notice that the results from the indicated experiment were used in two figures. C, S and SL stands for control, Snail1-transfected, and Snail1- and LEF1-NAT-tranfected HT29 M6 cells, respectively. The part boxed in yellow (control and Snail1-transfected) was presented in Fig 1G; the part boxed in red (Snail1-transfected and Snail1-transfected plus NAT), in Figure 3B.
